# Web-Based Versus Print-Based Physical Activity Intervention for Community-Dwelling Older Adults: Crossover Randomized Trial

**DOI:** 10.2196/32212

**Published:** 2022-03-23

**Authors:** Claudia R Pischke, Claudia Voelcker-Rehage, Tiara Ratz, Manuela Peters, Christoph Buck, Jochen Meyer, Kai von Holdt, Sonia Lippke

**Affiliations:** 1 Institute of Medical Sociology, Centre for Health and Society, Medical Faculty Heinrich Heine University Duesseldorf Duesseldorf Germany; 2 Department of Neuromotor Behavior and Exercise, Institute of Sport and Exercise Sciences University of Muenster Muenster Germany; 3 Department of Psychology and Methods Jacobs University Bremen Bremen Germany; 4 Department of Prevention and Evaluation Leibniz Institute for Prevention Research and Epidemiology – BIPS Bremen Germany; 5 Department of Biometry and Data Management Leibniz Institute for Prevention Research and Epidemiology – BIPS Bremen Germany; 6 OFFIS – Institute for Information Technology Oldenburg Germany

**Keywords:** physical activity, older adults, eHealth, print-based intervention, web-based intervention, physical activity promotion, healthy aging, preferences, randomized trial, mobile phone

## Abstract

**Background:**

Fewer than half of older German adults engage in the recommended levels of endurance training.

**Objective:**

The study aim is to compare the acceptance and effectiveness of two interventions for physical activity (PA) promotion among initially inactive community-dwelling older adults ≥60 years in a 9-month, crossover randomized trial.

**Methods:**

Participants were recruited in person and randomized to one of the following interventions for self-monitoring PA: a print-based intervention (PRINT: 113/242, 46.7%) or a web-based intervention (WEB: 129/242, 53.3%). Furthermore, 29.5% (38/129) of those in the web-based intervention group received a PA tracker in addition to WEB (WEB+). After randomization, the participants and researchers were not blinded. The participants’ baseline intervention preferences were retrospectively assessed. All the intervention groups were offered 10 weekly face-to-face group sessions. Afterward, participants could choose to stay in their group or cross over to one of the other groups, and group sessions were continued monthly for another 6 months. 3D accelerometers to assess PA and sedentary behavior (SB) at baseline (T0), 3-month follow-up (T1), and 9-month follow-up (T2) were used. Adherence to PA recommendations, attendance of group sessions, and intervention acceptance were assessed using self-administered paper-based questionnaires. Linear mixed models were used to calculate differences in moderate to vigorous PA (MVPA) and SB between time points and intervention groups.

**Results:**

Of the 242 initially recruited participants, 91 (37.6%) were randomized to the WEB group; 38 (15.7%) to the WEB+ group; and 113 (46.7%) to the PRINT group. Overall, 80.6% (195/242) of the participants completed T1. Only 0.4% (1/242) of the participants changed from the WEB group to the PRINT group and 6.2% (15/242) moved from the PRINT group to the WEB group (WEB-WEB: 103/249, (41.4%); PRINT-PRINT: 76/249, 30.5%) when offered to cross over at T1. Furthermore, 66.1% (160/242) of participants completed T2. MVPA in minutes per day increased between baseline and T1, but these within-group changes disappeared after adjusting for covariates. MVPA decreased by 9 minutes per day between baseline and T2 (β_time_=−9.37, 95% CI −18.58 to −0.16), regardless of the intervention group (WEB vs PRINT: β_group*time_=−3.76, 95% CI −13.33 to 5.82, WEB+ vs PRINT: β_group*time_=1.40, 95% CI −11.04 to 13.83). Of the participants, 18.6% (38/204) met the PA recommendations at T0, 16.4% (26/159) at T1, and 20.3% (28/138) at T2. For SB, there were no significant group differences or group-by-time interactions at T1 or T2. Intervention acceptance was generally high. The use of intervention material was high to moderate at T1 and decreased by T2.

**Conclusions:**

There was little movement between intervention groups at T1 when given the choice, and participation was not associated with increases in PA or decreases in SB over time.

**Trial Registration:**

German Clinical Trials Register DRKS00016073; https://www.drks.de/drks_web/navigate.do?navigationId=trial.HTML&TRIAL_ID=DRKS00016073

## Introduction

### Background

Engaging in an active lifestyle with regular physical activity (PA) [1] is associated with higher physical, cognitive, and functional health across the life course [1,2], and web-based support can help individuals to adopt and maintain PA [3]. At the time of study conception, the World Health Organization (WHO) and the American College of Sports Medicine recommended that adults aged 18-64 years, as well as those aged ≥65 years, should perform moderate to vigorous endurance training for at least 150 minutes per week (in 10-minute bouts) [4]. Furthermore, performing flexibility, strength, and balance exercises twice per week is recommended [5,6]. The 2020 update of the recommendations included several modifications. For example, the authors of the update state that “all adults should undertake 150-300 min of moderate-intensity, or 75-150 min of vigorous-intensity physical activity, or some equivalent combination of moderate-intensity and vigorous-intensity aerobic physical activity, per week” [7]. An additional change is that 10-minute bouts of PA are no longer deemed relevant. Instead, bouts of moderate to vigorous PA (MVPA) of any duration count, taking new evidence into account, which suggests that the total PA volume is more important than bouts. Furthermore, high-certainty evidence summarized for the development of the new guidelines indicates that balance and functional exercises are relevant for maintaining physical function and reducing falls [7]. Hence, in the update for the age group of ≥65 years, the recommendation is to incorporate these types of exercises at moderate or greater intensity on 3 or more days per week in existing routines [7].

Less than half of the German adults aged ≥65 years meet the former recommendations for endurance training (42%), and only one-third meet the strength training recommendations [6]. However, compared with the European Union average of adults in this age segment (26.2% of women and 35.7% of men reach the recommendation of 150 minutes of MVPA per week), German men and women display slightly higher proportions of adults reaching the recommendations (45.5% and 51.2% for women and men, respectively) [8]. Furthermore, results based on the European Health Interview Survey and the Survey of Health Aging and Retirement in Europe examined associations between the proportion of European adults >65 years, reaching the recommendation of >150 minutes of PA per week and the proportion of prefrail or frail individuals suggests a negative association [9]. To prevent frailty in older adults, Haider et al [9] called for “community-based approaches aimed at achieving PA recommendations” at the population level and the creation of built environments enabling PA [9]. Previous research conducted in Germany indicated that population-based approaches to increase PA, such as mass media campaigns, community-based multicomponent interventions, and environmental approaches, can be effective in the general population [10]. In addition, individual-level interventions provide opportunities to further increase the effect of such population-level approaches [1,11-13]. However, the role of different modalities in delivering these intervention approaches to older adults remains unclear.

The results of several systematic reviews indicate that participation in interventions providing information on PA face-to-face or via printed materials leads to increased PA levels in older adults [14-16]. Engagement in web-based PA interventions is also associated with increased MVPA, walking, and a higher daily step count in the intervention group than in the control group [17,18]. Furthermore, the results of a systematic review evaluating the effectiveness of eHealth interventions compared with non-eHealth interventions or no intervention in adults ≥55 years suggest that eHealth interventions can effectively promote PA in the short term [13], but there is still a lack of evidence regarding long-term effects. Recent evidence from a review examining the effects and characteristics of PA promotion interventions aimed at community-dwelling adults >50 years indicates that increases in PA can be sustained for up to 12 months [19]. In conclusion, it is still unclear whether eHealth interventions have a greater impact on PA behavior than non-eHealth (eg, print-based) interventions in adults who are ≥60 years and whether increased levels of PA can be maintained over longer periods.

Furthermore, the influence of individual preferences for intervention modality and variances in the impact on intervention outcomes is still not well understood [20,21]. Previous studies suggest that preferences may vary by age, sex, BMI, or social or living environment [15,22,23]. For example, preference for a web-based intervention was positively related to younger age [22,23] and high internet use and was negatively associated with the female sex. Conversely, older women with obesity were more likely to choose print-based interventions [22]. These variations in sociodemographic characteristics may also explain the differences in the use of PA trackers [24]. To increase the impact of this tool that has already been shown to be effective [25], the use of trackers in PA interventions should be aligned with preferences of different target groups [15]. Both retention in intervention studies and adherence to intervention components may improve if individual preferences for interventions are considered [12,15]. Hence, in this study, a crossover design was used to examine the role of personal preferences for different delivery modes in intervention effectiveness.

This study (PROMOTE II) was funded by the Federal Ministry of Education and Research (Bundesministerium für Bildung und Forschung), as part of the Physical Activity and Health Equity: Primary Prevention for Healthy Aging research network [26]. It builds on the results of a previous study embedded in the network (PROMOTE I; [27-29]), which tested the effectiveness of 2 tailored web-based interventions for the promotion of a physically active lifestyle in adults aged 65-75 years in a community-based intervention trial against a delayed-intervention control group. In a previous study, we found relatively high baseline PA levels in the intervention participants. On the basis of this observation, individuals who had been physically active regularly for at least 2.5 hours per week for >1 year were excluded from this study. Furthermore, study dropout was higher in the group assigned to use PA trackers in addition to a website than in the website-only group, indicating that randomization to a modality that was not a preference led to participants deciding to quit the intervention [27-29].

### Objectives

On the basis of these results gathered in the preceding study, this study included the following four aims:

1. To adapt and simplify the web-based intervention of a previous study to further improve usability and develop a simple print-based intervention that initially inactive participants with little affinity to technology find easy to use.
2. To investigate the acceptance and use of two interventions (web- vs print-based) and changes in PA among older adults (≥60 years) in a crossover randomized trial over the course of 9 months.
3. To examine the role of personal preferences for different delivery modes in intervention effectiveness.
4. To explore the associations between changes in PA and possible changes in physical fitness and cognitive capacity in a pooled sample of participants in both PROMOTE I and II trials.

In this paper, we report the results of the first 3 study aims. The results addressing the last aim will be reported in a subsequent paper. We hypothesized that both interventions would significantly increase MVPA and decrease sedentary behavior (SB) at the first and second follow-ups [30].

## Methods

### Participants and Procedures

#### Recruitment

A random sample of 3492 adults aged ≥60 years from 14 districts in Bremen, Germany, were invited to participate in the study via mail. The names and addresses were provided by the residents’ registration offices. This included individuals who resided in districts that met the following requirements:

1. Districts that were not part of the municipalities targeted in PROMOTE I
2. Districts that were in close proximity to the two study centers (one in the Northwest and one in the Northeast of the city of Bremen, Germany)
3. Districts where the project team had already established previous liaisons, including contacts with stakeholders facilitating community involvement during the implementation of the intervention

Reminders were sent out after 2 weeks in cases of no response. The study was also publicized in local newspaper articles and mentioned during talks of the research staff, sparking the interest of 168 individuals who called up the research team directly and were consequently screened for eligibility. Eligibility for study participation was determined through computer-assisted telephone interviews with trained study nurses following the inclusion and exclusion criteria outlined below. The sample size and power calculations are described in detail in a previously published study protocol [30].

#### Ethical Approval and Informed Consent

The study obtained ethical approval from the Medical Association of Bremen, Germany, on July 3, 2018 (RA/RE-635). The study was registered at the German Clinical Trials Register on January 10, 2019 (DRKS00016073). Potential participants were informed of the study during the initial telephone interviews and were fully informed during an introductory face-to-face briefing session and were requested to provide informed consent. They were also told that they would be randomized to one of the intervention groups and knew about their existence. At the end of the introductory session, all participants were fully informed of the study and provided informed consent. The participants, research staff conducting the study, or statistician analyzing the data were not blinded to the intervention.

#### Inclusion and Exclusion Criteria

Briefly, individuals were included in the study if they were aged ≥60 years, lived independently, and provided informed consent. Individuals were excluded from the study if they reported that they had been physically active regularly for at least 2.5 hours per week for >1 year. Furthermore, having participated in the previous trial, a planned vacation during the intervention period exceeding 2 weeks, a medical condition or diagnosis prohibiting PA, severe visual or other impairments, implanted cardiac devices, or occasional syncopal episodes led to exclusion (see the study protocol by Pischke et al [30] for further details). Cognitive state was measured using the Mini-Mental State Examination 2–brief version (MMSE-2-BV) [31], and the exclusion criterion was initially set to an MMSE-2-BV score of ≤14. As the manual for the MMSE-2-BV does not define a cutoff value for the determination of cognitive impairment, the initially chosen cutoff value was re-evaluated during the study and was found to be too conservative. On the basis of previous studies [32,33], the cutoff value was adapted, and individuals with an MMSE-2-BV score <13 were excluded.

#### Randomization and Allocation

**Figure 1:**
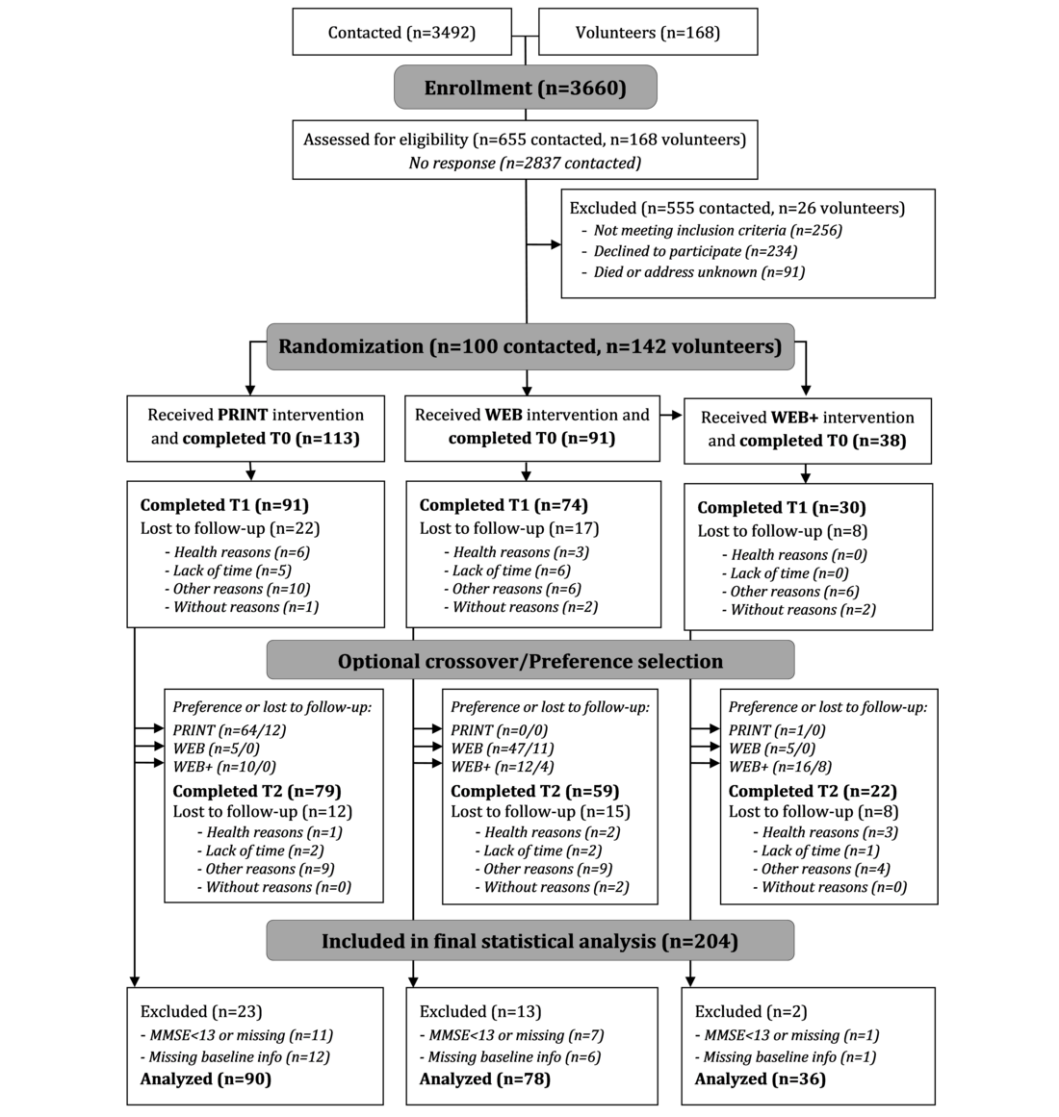
Participant flow. MMSE: Mini-Mental State Examination; T0: baseline assessment; T1: 3-month follow-up; T2: 9-month follow-up.

Of the 3660 older adults invited, 823 (22.49%) individuals were assessed for eligibility during computer-assisted telephone interviews (Figure 1). In total, 70.6% (581/823) of the potential participants were excluded. After determination of eligibility, 29.4% (242/823) of the study participants were randomized to one of two groups by the study nurse applying an allocation ratio of 1 to 1: (1) a print intervention with subjective PA self-monitoring via printed PA-pyramid (PRINT: 113/242, 46.7%) and (2) a web-based intervention with subjective PA self-monitoring via a web-based PA-pyramid (WEB: 129/242, 53.3%). Furthermore, 29.5% (38/129) of those in the web-based intervention group were randomly selected and received a PA tracker (objective PA self-monitoring) in addition (WEB+ group). Weekly time slots were randomly assigned to the 3 intervention groups. The first 30% of the timeslots reserved for the WEB group received Fitbit devices (WEB+). Participants were blinded to the intervention group during randomization (ie, they were free to choose from available time slots during the telephone interview with the study nurse, without knowing which intervention group they were assigned to).

### Interventions

**Figure 2:**
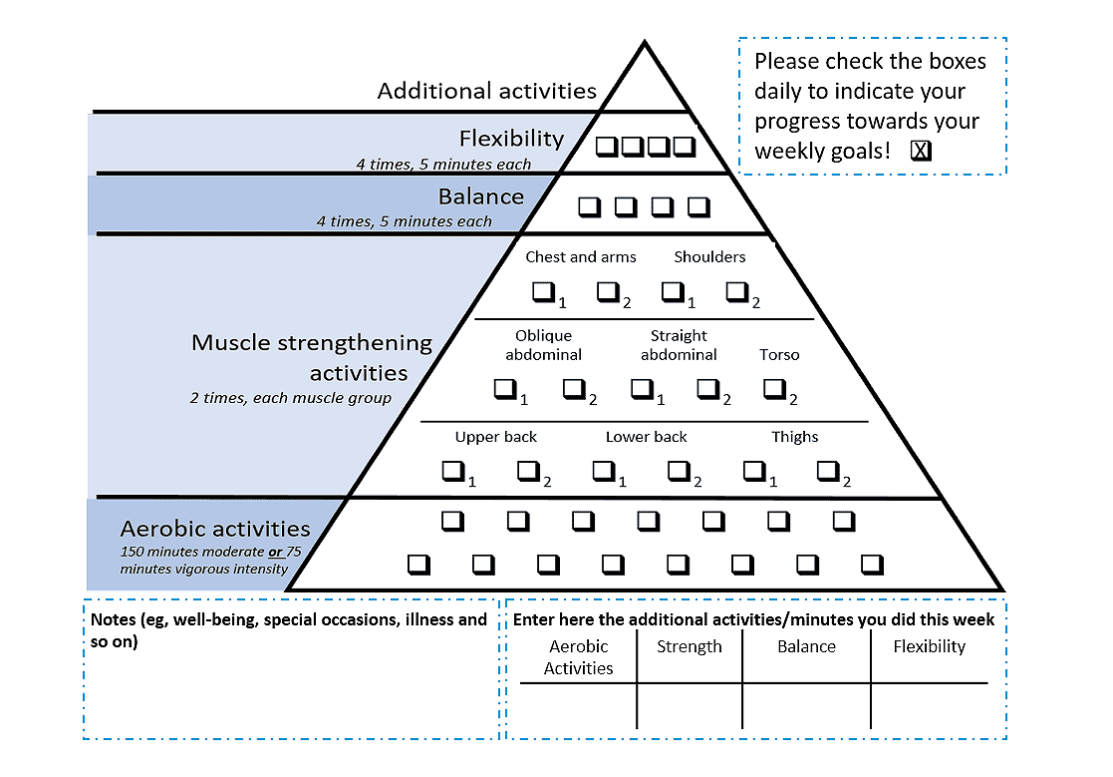
Intervention material (PRINT).

The design process of the interventions and their contents are described elsewhere [30]. They were designed based on the state-of-the-art research on PA and the results of focus group discussions conducted with the target group. The intervention content was based on self-regulation theory and various behavior change techniques facilitating regular self-monitoring of PA [34,35]. Participants in both groups received PA recommendations according to the WHO, and brochures (web based and in print) were provided outlining exercises for different difficulty levels, showing pictures of male versus female older adults modeling these exercises [30]; they additionally received a diary to track their PA. Depending on the group assignment, all intervention materials were either provided as printed materials or made available on the website. The smartphone app additionally provided access to the exercises and PA diary for individuals in the WEB and WEB+. On the website, in the Android web app, as well as in the printed diary, weekly feedback regarding whether PA goals were reached was provided (Figure 2), the number of minutes or units exercised and the units required to reach the goal were displayed. The WEB+ group used a PA tracker (Fitbit Zip, Fitbit Inc) in addition to the website or app, and the daily step count tracked with the device was synchronized with the website. No prompts or reminders were used on the website or in the app.

In tandem with the 10-week PRINT or WEB and WEB+ interventions, all 3 intervention groups were offered weekly face-to-face group sessions (facilitated by trained student assistants) with up to 25 participants per group, who were encouraged to attend the sessions. The 90-minute group sessions included performing the exercises in groups and going for joint walks and discussing weekly health education topics, and the participants were encouraged to ask open questions regarding the exercises (also see the study protocol by Pischke et al [30]). During their first weekly group meeting, participants received the necessary equipment (printed material or access information for the website and a Fitbit device) and a comprehensive introduction on how to use the equipment and materials. After 10 weeks, group meetings were continued monthly for another 6 months. After the last weekly group sessions, participants chose to continue using the material from their intervention group or to start using material from one of the other groups (Figure 1).

### Outcomes and Measures

#### Data Collection

Two weeks before the intervention started, at the introductory event, participants received the questionnaires for the baseline assessment (T0) and were instructed on how and when to wear the accelerometer to measure their baseline PA behavior. They were asked to bring this data collection material to their first weekly group session, where they completed a short version of the MMSE-2-BV, which was conducted individually in a separate room by research staff [31]. During the first sessions of the 3- and 9-month follow-ups (T1 and T2), study participants received the data collection material in person from the research staff and were asked to send it back within 1 week via mail.

#### Sociodemographic and Baseline Variables

Table 1 presents the outcome measures, validated instruments, and assessment times. Sociodemographic information (eg, age, sex, family status, and employment) was collected via self-administered questionnaires at baseline as summarized in Table 1. Need-weighted household income per capita was derived from the number of individuals living in the household and the monthly household income according to the German Microcensus [36]. The variable was then classified into low-, middle-, and high-income households. Education level was coded using the 2011 version of the International Standard Classification of Education (ISCED). Individuals with higher educational status received higher scores (range 1-8) [37]. The variable was dichotomized into low or medium level of education (ISCED score 1-4) and high level of education (ISCED score 5-8). BMI was calculated based on the self-reporting of height (T0) and weight (assessed at all time points) and dichotomized into underweight or normal weight and overweight or obese according to the WHO BMI classification for adults aged ≥20 years [38]. In addition, neighborhood, subjective general health (excellent or very good, good, less good, or poor), activity-related social support by family and friends, technology readiness (technology acceptance), technology competence beliefs (consisting of acceptance, competence belief, technology control belief, and technology willingness or readiness), and ownership and frequency of use of digital devices were measured.

**Table 1:** Selected measures in the self-administered study questionnaire used in the analysis for this study.

Outcome measure	Instrument or scale^a^	Time of assessment
Sociodemographic information (sex, age, education, family status, employment status, and household income)	German Health Interview and Examination Survey for Adults, questionnaire for assessing seniors’ demographic and sociostructural data in Germany	T0^b^
Height and weight	Self-generated items	T0
Physical activity and neighborhood environment	Physical activity neighborhood environment scale	T0
Walking environment	Neighborhood Scales, walking environment, 1 Item (activity friendly score)	T0
Social support for engaging in physical activity	Activity-related support by family and friends (modified) and activity-related social support	T0, T1,^c^ T2^d^
Subjective health status	Short-Form–12, 1 item	T0, T1, T2
Technology commitment	Technology commitment scale	T0, T1, T2
Technology use and experience	Self-generated items	T0, T1, T2
Use and acceptance of various components of the interventions (website and printed material), attendance of the offered group sessions, and overall satisfaction with the interventions	Self-generated items	T1, T2
Preference regarding intervention material at baseline (retrospective)	Self-generated items	T2
Reasons for crossing over or not crossing over after 3 months	Self-generated items	T2

^a^References for the instruments can be found in the study protocol [30].

^b^T0: baseline assessment.

^c^T1: 3-month follow-up.

^d^T2: 9-month follow-up.

#### PA and SB Outcomes

The main outcomes were MVPA and SB in minutes per day assessed at T0, T1, and T2 using triaxial accelerometers (GT3x+ [ActiGraph]). Participants were instructed to wear the accelerometer at the right hip over a course of 7 days for 24 hours. Accelerometer data were processed using the Actilife 6.8.0 software (ActiGraph) and R (version 3.6.1; R Foundation for Statistical Computing) [39] was used to identify nonwear times and classify PA levels into the categories described below.

Valid wear time was derived using the wear- and nonwear time classification algorithm by Choi et al [40], using a 90-minute window of consecutive zeros allowing a 2-minute interval of nonzero counts, and valid days were defined as having at least 8 hours (480 minutes) of valid wear time. There had to be at least three valid days available for each participant, including 1 weekend day, for the analysis. Using 1-second epochs, counts were categorized into SB (0-99 counts per minute [cpm]), as well as light (0-2690 cpm), moderate (2691-6166 cpm), vigorous (6167-9642 cpm), moderate to vigorous (2691-9642 cpm), and highly vigorous (>9642 cpm) PA, according to Sasaki et al [41], considering the vector magnitude.

The daily minutes for MVPA and SB were determined by dividing the total minutes by the number of days the accelerometer was worn. SB was additionally calculated in bouts of at least 30 minutes, and time spent with MVPA was calculated in bouts of at least 10 minutes. Minutes per week for MVPA and SB in the mentioned bouts were derived by multiplying the daily average minutes in 10-minute or 30-minute bouts, respectively, by 7. Furthermore, minutes of MVPA per week in bouts of 10 minutes was dichotomized as meeting the WHO recommendation (≥150 minutes per week of MVPA in bouts of at least 10 minutes) or not meeting them. The season during the accelerometer measurement was derived from the date of examination and categorized into autumn or winter for the months of October to February and spring or summer for the months of March to September.

#### Adherence, Use, and Acceptance

Information on acceptance of the group sessions and intervention material was assessed with self-generated items (eg, frequency of general use, use of different components [on a 5-point Likert scale ranging from *never* to *daily*], and perceived helpfulness of intervention components [on 5-point Likert scales ranging from *not helpful at all* to *very helpful*]). The reasons for dropping out of the study and for crossing or not crossing over to the other intervention groups and preferences for intervention material were also assessed (Table 1).

### Statistical Analyses

Descriptive statistics, that is, mean, SD, range, or proportion, were calculated to describe the study characteristics across intervention groups and surveys. The effects of time, group, and time by group on MVPA and SB, either in average minutes per day or minutes in bouts per week, were examined using multivariate linear mixed models that can handle unbalanced longitudinal data with varying numbers of repeated measurements per participant [42]. Analyses were adjusted for sex, age, BMI classification, level of education, family status, employment status, household income, subjective health status, built environment, activity-related support, preference, season, and valid wear time. Model diagnostics, such as residual plots and *Q*–*Q* plots, were used to check the assumptions of the linear mixed models. No violation of the assumptions of the linear mixed models was observed. In addition, outliers were checked and found to be unproblematic.

As cell counts for crossover groups were very small, linear mixed models were not run for the potential crossover combinations at the 3-month follow-up. The analyses regarding the intervention groups in this study were calculated using the group allocation at baseline as the indicator of the intervention group (ie, all analyses were conducted using the originally assigned groups). Only the numbers and proportions of individuals in the crossover combinations were reported descriptively. In addition, information assessed at follow-up (eg, preferences and reasons for crossing over or not crossing over to the other mode of delivery) and indicators of intervention adherence and acceptance were calculated. All statistical analyses were performed using SPSS 26 (IBM) [43] and SAS 9.4 [44], where the GLIMMIX procedure was used particularly for linear mixed modeling.

## Results

### Participant Flow and Baseline Characteristics

Of the 3660 older adults invited, 823 (22.49%) individuals were assessed for eligibility during computer-assisted telephone interviews (Figure 1). Of the 242 initially recruited participants, 91 (37.6%) were randomized to the WEB group; 38 (15.7%), to the WEB+ group; and 113 (46.7%), to the PRINT group. After 3 months, 80.6% (195/242) of the participants completed T1 (from the original group allocation; WEB: 74/91, 81%; WEB+: 30/36, 83%; and PRINT: 91/113, 80.5%). After T1, 91.8% (179/195) of the participants chose to remain in their previous intervention group, and 8.2% (16/195) decided to crossover to the other group. Finally, 66.1% (160/242) of participants completed T2 (from the original group allocation; WEB: 59/91, 65%; WEB+: 22/38, 58%; and PRINT: 79/113, 69.9%). Attrition rates from baseline to T2 were 33.9% across the groups (WEB: 35.2%, WEB+: 42.1%, and PRINT: 30.1%).

Observations from participants (T0, T1, and T2) were excluded from the analysis if they were missing information on BMI (14/501, 2.8%), subjective health, family status, or education (34/501, 6.8%), and if the MMSE-2-BV score was <13 (51/501, 10.2%). In total, 501 observations from 204 participants were included in the analysis (PRINT: 90/204, 44.1%; WEB: 78/204, 38.2%; and WEB+: 36/204, 17.7%). For follow-up samples, the exclusion criteria reduced the sample sizes to 159 at T1 and 138 at T2.

The baseline demographic characteristics of the participants included in the analysis are shown in Multimedia Appendix 1. Overall, the mean age was 68.7 (SD 5.4, range 60-82) years, with a slightly higher average age in the WEB+ group (70.5, SD 6.0 years). Fewer than half of the participants (87/204, 42.6%) had a BMI in the underweight or normal weight range according to the WHO standards. Across all groups, except for the WEB+ group, women were overrepresented. The proportion of female participants slightly differed among the study groups (PRINT: 75%, WEB: 64%, and WEB+: 47%). In the total sample of 204 participants, 112 (54.9%) had a high level of education, 110 (53.9%) were married, and 136 (66.7%) and 30 (14.7%) reported good and very good health, respectively. Participants rated their acceptance as average (mean 2.7, SD 0.83), and their competence beliefs (mean 3.9, SD 0.83), control beliefs (mean 3.9, SD 0.77), and overall willingness to deal with new technologies (mean 3.5, SD 0.63) with stronger agreement. The recommended level of MVPA was 12% (11/90) of the participants in the PRINT group, 23.1% (18/78) of the participants in the WEB group, and 25% (9/36) of the participants in the WEB+ group. Baseline differences were accounted for by including relevant variables as covariates in linear mixed models.

We analyzed potential selectivity by calculating Cohen *d* using the mean difference and pooled SD between the recruited and analyzed samples for continuous baseline characteristics. Cohen *h* was calculated based on the proportions of categorical baseline characteristics [45]. The analysis sample (n=204) did not differ from the recruited sample (n=242) in baseline characteristics as the effect sizes (Cohen *d* and *h*, respectively) were all <0.20. The only exception was the cognitive state: the analysis sample had a slightly higher MMSE-2-BV mean score compared with the recruited sample (Cohen *d*=0.22). This was expected because of the exclusion criteria (Figure 1).

### PA and SB Outcomes

Overall, the proportion of individuals reaching the MVPA recommendation did not change over time; 18.6% (38/204) of them reached the WHO recommendation at baseline: 16.3% (26/159) at T1 and 20.2% (28/138) at T2 (Multimedia Appendix 2). In all 3 intervention groups, MVPA in minutes per day seemed to increase between baseline and T1: from 84.4 (SD 33.0) to 92.3 (SD 31.5) minutes in WEB, from 84.4 (SD 39.7) to 95.5 (SD 37.7) minutes in WEB+, and from 82.8 (SD 29.2) to 84.3 (SD 26.1) minutes in PRINT (Table 2). When adjusting for covariates, the least squares mean differences in time between baseline and T1 within the intervention groups were not significant. There was a significant decrease between baseline and T2 in the whole study sample by 9 minutes of MVPA per day (β_time_=−9.37, 95% CI −18.58 to −0.16). Within the groups, the least squares mean decrease between baseline and T2 was significant for WEB (mean difference −13.12, 95% CI −23.40 to −2.84) and PRINT (mean difference −9.37, 95% CI −18.58 to −0.16; Table 2). Compared with PRINT, there were no significant group differences and group-by-time interactions at T1 or T2 (Table 2). Compared with PRINT and baseline, the WEB group at T2 was approximately 4 minutes per day less active in MVPA (β_group*time_=3.76, 95% CI −13.33 to 5.82) and the WEB+ group at T2 was approximately 1 minute per day more active in MVPA (β_group*time_=1.40, 95% CI −11.04 to 13.83).

**Table 2:** Results of the linear mixed models (time, group, intervention effects, and comparison of intervention effects) for moderate to vigorous physical activity (MVPA; minutes per day and 10-minute bouts).^a^

Characteristics			Indicators per time point, mean (SD)							Difference in time within group (reference T0^b^), least squares mean (95% CI)					Time difference (reference T0), β (95% CI)					Group difference (reference PRINT), β (95% CI)			Group-by-time interaction (reference PRINT at T0), β (95% CI)		
			T0		T1^c^		T2^d^		T1			T2		T1			T2					T1			T2
**MVPA (minutes per day)**																									
	WEB	84.4 (33.0)		92.3 (31.5)		81.3 (31.6)		2.68 (−6.43 to 11.78)			−13.12 (−23.40 to −2.84)		−1.46 (−9.87 to 6.95)			−9.37 (−18.58 to −0.16)		4.90 (−4.49 to 14.30)			4.13 (−4.69 to 12.96)			−3.76 (−13.33 to 5.82)	
	WEB+	84.4 (39.7)		95.5 (37.7)		90.0 (36.7)		1.98 (−9.90 to 13.87)			−7.97 (−20.26 to 4.32)		N/A^e^			N/A		8.73 (−3.44 to 20.89)			3.44 (−7.74 to 14.62)			1.40 (−11.04 to 13.83)	
	PRINT	82.8 (29.2)		84.3 (26.1)		80.8 (28.8)		−1.46 (−9.87 to 6.95)			−9.37 (−18.58 to −0.16)		N/A			N/A		N/A			N/A			N/A	
**MVPA in 10-minute bouts (minutes per week)**																									
	WEB	89.1 (121.2)		79.1 (106.6)		78.2 (108.4)		−17.06 (−57.94 to 23.83)			−49.64 (−95.83 to −3.45)		−2.28 (−40.18 to 35.62)			−37.7 (−78.87 to 3.48)		18.51 (−16.50 to 53.52)			−14.78 (−55.32 to 25.77)			−11.94 (−55.96 to 32.08)	
	WEB+	98.3 (139.5)		119.8 (165.3)		86.2 (130.0)		15.76 (−38.31 to 69.83)			−53.48 (−109.47 to 2.51)		N/A			N/A		24.54 (−20.81 to 69.89)			18.04 (−33.38 to 69.45)			−15.78 (−72.90 to 41.34)	
	PRINT	76.9 (108.9)		70.0 (87.1)		73.3 (81.0)		−2.28 (−40.18 to 35.62)			−37.7 (−78.87 to 3.48)		N/A			N/A		N/A			N/A			N/A	

^a^The linear mixed model was adjusted for age, sex, BMI, level of education, family status, employment status, household income, subjective health status, built environment, activity-related support, preference, season, and valid wear time.

^b^T0: baseline assessment.

^c^T1: 3-month follow-up.

^d^T2: 9-month follow-up.

^e^N/A: not applicable.

With regard to MVPA in 10-minute bouts in minutes per week, only the WEB+ group seemed to be more active at T1 with mean 119.8 (SD 165.3) minutes compared with baseline with mean 98.3 (SD 139.5) minutes (Table 2). However, the estimated difference in time within WEB+ was not significant (least squares mean difference 15.76, 95% CI −38.48 to 69.83). At T2, there was a significant estimated mean decrease in minutes of MVPA in 10-minute bouts per week within the WEB (least squares mean difference −49.64, 95% CI −95.83 to −3.45). Compared with PRINT, there were no significant group differences and group-by-time interactions at T1 or T2 (Table 2).

For sedentary time in 30-minute bouts in minutes per week, there was a significant estimated decrease at T1 within the WEB group (mean difference −212.00, 95% CI −422.14 to −3.84). However, the CI was very wide and the effect was not maintained until T2 (Table 3). Compared with the PRINT group, there were no significant group differences and group-by-time interactions at T1 or T2 (Table 3). At T2, there were no significant intergroup differences over time.

At all 3 time points, the mean sedentary time per day was between 600 and 700 minutes, that is, between 10 and 11.5 hours (minimum 420 minutes, maximum 1200 minutes). There were no significant within-group differences at T1 and T2 with regard to sedentary time in minutes per day after adjusting for covariates. There were no significant group differences or group-by-time interactions at T1 or T2 (Table 3). There were no significant within-group differences over time at T2 (β_time_=5.29, 95% CI −9.12 to 19.69). Compared with PRINT and baseline groups, the WEB group at T2 spent approximately 10 more minutes per day with sitting (β_group*time_=10.41, 95% CI −4.49 to 25.31) and the WEB+ group at T2 spent approximately the same minutes per day with sitting (β_group*time_=−0.13, 95% CI −19.49 to 19.22; Table 3).

**Table 3:** Results of the linear mixed regression models (time, group, intervention effects, and comparison of intervention effects) for sedentary behavior (minutes per day and 30-minute bouts).^a^

Characteristics		Indicators per time point, mean (SD)				Difference in time within group (reference T0^b^), least squares mean (95% CI)			Time difference (reference T0), β (95% CI)			Group difference (reference PRINT), β (95% CI)		Group-by-time interaction (reference PRINT at T0), β (95% CI)	
		T0	T1^c^	T2^d^	T1		T2	T1		T2			T1		T2
**Sedentary time (minutes per day)**															
	WEB	630.2 (102.2)	633.2 (90.9)	638.1 (88.2)	−5.20 (−19.43 to 9.02)		15.7 (−0.36 to 31.76)	1.04 (−12.09 to 14.17)		5.29 (−9.12 to 19.69)	−5.88 (−21.14 to 9.39)		−6.24 (−19.98 to 7.49)		10.41 (−4.49 to 25.31)
	WEB+	637.7 (74.5)	642.6 (79.0)	628.9 (94.1)	−10.83 (−29.36 to 7.69)		5.15 (−14.00 to 24.31)	N/A^e^		N/A	−2.53 (−22.31 to 17.25)		−11.88 (−29.27 to 5.52)		−0.13 (−19.49 to 19.22)
	PRINT	639.0 (78.3)	649.9 (128.5)	646.8 (120.6)	1.04 (−12.09 to 14.17)		5.29 (−9.12 to 19.69)	N/A		N/A	N/A		N/A		N/A
**Sedentary time in 30-minute bouts (minutes per week)**															
	WEB	2228.1 (905.2)	2098.7 (851.1)	2348.2 (731.5)	−212.99 (−422.14 to −3.84)		179.25 (−56.85 to 415.35)	−18.83 (−211.94 to 174.29)		72.31 (−139.36 to 283.98)	−107.81 (−327.33 to 111.70)		−194.16 (−396.46 to 8.14)		106.94 (−112.63 to 326.51)
	WEB+	2368.4 (839.3)	2336.5 (785.7)	2402.2 (701.7)	−160.20 (−432.82 to 112.43)		72.65 (−209.29 to 354.59)	N/A		N/A	−16.37 (−300.69 to 267.95)		−141.37 (−397.69 to 114.96)		0.34 (−284.79 to 285.46)
	PRINT	2301.3 (798.9)	2371.8 (960.6)	2402.1 (900.5)	−18.83 (−211.94 to 174.29)		72.31 (−139.36 to 283.98)	N/A		N/A	N/A		N/A		N/A

^a^The linear mixed model was adjusted for age, sex, BMI, level of education, family status, employment status, household income, subjective health status, built environment, activity-related support, preference, season, and valid wear time.

^b^T0: baseline assessment.

^c^T1: 3-month follow-up.

^d^T2: 9-month follow-up.

^e^N/A: not applicable.

### Attendance, Use, and Acceptance of Intervention Components

Overall, attendance of the face-to-face components of the intervention was high, with an average of 8/10 weekly group sessions attended and 2/3 monthly group sessions attended (Multimedia Appendix 3). Regarding the use of the PA diary, there were no marked differences among the intervention groups at T1. The exercise brochure was used at least once per week or daily by 68% (50/73), 48% (29/60), and 38% (10/26) of the PRINT, WEB, and WEB+ groups, respectively. The overall use of intervention material was high to moderate at T1 (the PA diary was used by between 65% (17/26) and 78% (47/60) of the participants at least once per week or daily) and declined by T2. At T2, approximately 44% (24/55) of the participants in the PRINT group, 49% (23/47) in the WEB group, and 58% (19/36) in the WEB+ group still used the PA diary at least once per week or daily. The use of the smartphone app was very low in the WEB group but higher in the WEB+ group.

Acceptance of the interventions was generally high; approximately half of the participants agreed that the program was at least somewhat helpful for being physically active (T1), and stated that they would recommend it to others (T1; Multimedia Appendix 3). Retrospectively, between 73% (49/55) and 78% (39/47) of the participants in each group stated that their random allocation matched their initial preference (T2; Multimedia Appendix 1). The most commonly reported reasons for crossing over were “wanted to try something new,” “liked the website and wanted to use the fitness tracker in addition,” and “high affinity to technology.” The reason for not crossing over reported most often was “completely satisfied with the current material.” Further reasons listed included “did not want to lose contact to the previous group members,” “printed version seemed impractical,” or “not technology-affine and wanted to keep the printed version.” No unintended effects were reported by the participants.

## Discussion

### Principal Findings

In summary, no intervention effects on MVPA were detected in this study, including 242 community-dwelling older adults aged ≥60 years who participated in a 9-month crossover randomized trial. MVPA did not increase but decreased over time, regardless of which group the participants were randomized to. The proportion of participants meeting the WHO recommendations for MVPA remained relatively stable, with approximately one-fifth of the participants meeting the recommendations at all 3 assessment points. The use of the intervention materials decreased slightly over time. Regarding SB, all 3 intervention groups displayed a decreasing trend in this risk behavior over time. However, no significant intergroup differences were observed in this regard. Interestingly, however, there was an indication that the reduction was most pronounced in the WEB group, which had decreased sedentary time in 30-minute bouts in minutes per week from baseline to T1. However, this effect was not maintained at follow-up, and no significant time-by-group interactions were observed. This study adds to the current knowledge that the mode of delivery (PRINT vs WEB) did not appear to affect the acceptance and effectiveness of the intervention content. Both were comparable across the groups. Unfortunately, however, none of the intervention conditions displayed increases in PA over the course of 9 months (for a comparison with previous research, see the following section).

It is conceivable that the intervention effects in certain subgroups were masked in the primary analyses. In an exploratory analysis, the presence of unobserved subgroups was investigated with regard to the latent change trajectories of MVPA and SB [46]. Regarding MVPA, latent change trajectory analysis revealed an initially sufficiently active and an initially insufficiently active subgroup, both of which remained constant over time. Regarding SB, an initially highly sedentary subgroup and moderately sedentary subgroup were identified. Although the moderately sedentary subgroup experienced slight increases in sitting time, the initially highly sedentary subgroup experienced significant decreases in SB and significant increases in PA levels [46]. This may suggest that our interventions were particularly useful for older adults with high initial SB levels.

Second, we found that despite having had the opportunity to try out another condition at the 6-month follow-up, very few participants took advantage of it. In total, only approximately 7.8% (16/204) switched conditions at follow-up (1/17, 6% from WEB [including WEB+] to PRINT; 15/17, 88% from PRINT to WEB). Two-thirds of participants in each condition stated at follow-up that their random allocation had matched their initial preference, suggesting that intervention participants felt content with the condition (and use of materials) to which they were assigned. In addition, feeling part of a group during the sessions and not wanting to leave the group may have played a role. Furthermore, 10.3% (21/204) of the participants stated that they did not want to lose contact with their groups. In fact, overall satisfaction with the group sessions and attendance rates were very high and did not differ among the modes of delivery. To conclude, these results suggest that the participants did not see any reason for switching to another mode of delivery. However, another explanation for the lack of movement between conditions at T1 could be that study participants who were randomized to a specific condition either refused to participate in the study or possibly dropped out of the study early, because they felt dissatisfied with using the intervention materials assigned to them in the condition that they were randomized to. Future pragmatic trials combining a randomized controlled trial with 2 different intervention arms in which participants can self-select will be necessary to further investigate the questions regarding the role of individual preferences raised in this study.

### Strengths and Limitations

Despite the advantages of the study design applied in this project and the objective measurement of MVPA using accelerometers, this study had several limitations. First, there was no untreated or placebo control group. Second, the preference for a certain intervention delivery mode at baseline was only assessed retrospectively, which may have led to a recall bias. However, we chose to assess preferences retrospectively because we anticipated disappointment (and possibly study dropout); if an individual was not randomized to the intervention group that they preferred to be in at baseline. Another question that remains is whether the recruitment channel that participants were recruited via (ie, print media vs mailed invitations) played a role when deciding for or against an intervention condition at the 6-month follow-up. This will be the topic of a future study. Finally, we were unable to recruit the number of study participants to the study that we had aimed for [30], and loss-to-follow-up was relatively high. As we did not meet the intended goal regarding the sample size, our analyses were underpowered. This problem was addressed using linear mixed modeling. Another limitation of our study is that the primary outcome defined in this study was not a state-of-the-art recommendation at the time of study completion. Thus, participants may not have been sufficiently motivated to engage in activities amounting to >10 minutes because, on the website or using PRINT materials for self-monitoring PA, they could only complete the PA diary if the activities leveled up to 10-minute bouts. In addition, we were unable to quantify the individual intervention effects of the group sessions on PA. Finally, neither participants nor researchers were blinded to the conditions, design, and aim of the study.

Several issues potentially causing selection bias and high attrition were identified in our study. One of the exclusion criteria was that participants had to own a PC with internet access. It is possible that individuals without this equipment were disadvantaged because we could not provide the equipment to participate in the study to them. Another selection bias may be the appointments made available to them. In particular, individuals who were still employed found it difficult to keep the appointments assigned to them and may have dropped out consequently. Furthermore, as discussed previously, despite the exclusion criteria already meeting the recommendation of 150 minutes per week, we found that many participants had higher levels of PA than were reported while entering the study and did not feel sufficiently challenged by our intervention. All of these issues have been previously discussed in the context of individual studies and meta-analyses conducted on the topics of selection, retention, and dropout in behavior change trials [47-49].

### Comparison With Previous Work

Compared with previous research suggesting that eHealth interventions can effectively promote PA in older adults in the short term [1,11-13,50-53], we were not able to demonstrate intervention effects for participants in the WEB and WEB+ conditions, neither in the short term at 3 months nor in the longer term at 9 months. This is puzzling, as several recently published systematic reviews and meta-analyses [50-53] demonstrate the effectiveness of eHealth interventions, including mobile interventions [52], for improving PA levels (eg, mean steps per day and minutes of daily MVPA, weekly PA, and MVPA [50]) in predominantly healthy older adults. Similar to our study, acceptance of eHealth intervention approaches was high in the studies included in these systematic reviews and meta-analyses [50]; however, contrary to our attrition rate, studies included in [50] predominantly reported attrition below 20% [50].

Looking at the evidence for the lack of effectiveness of eHealth interventions only, Elavsky et al [52] reported that 12 out of 29 randomized controlled trials and 8 out of 21 trials reporting pre-post changes in PA did not find any significant increases in PA. However, most studies that did not demonstrate effects did not include print-based conditions as a comparison group but control groups not receiving an intervention [52]. Hence, they could not be compared with the results of our study. Furthermore, in our study, we did not observe any differential effects of the intervention modality (WEB or WEB+ vs PRINT). A systematic review conducted by Muellmann et al [13] included several studies comparing print- and web-based intervention arms with contradictory results. Two studies by Peels et al [54,55] revealed that print- and web-based interventions were equally effective in promoting PA at the 6-month follow-up, but at the 12-month follow-up, only participation in the print-based interventions was associated with significant changes in PA. Van Stralen et al [56-58] compared a web-based intervention to a no-intervention control group and print-based intervention. Contrary to the findings by Peels et al [54,55], participants receiving the print-based intervention did not display any increases in PA at the 12-month follow-up, whereas participants who received the web-based intervention did. The contents of both WEB and PRINT interventions in our study were very similar and according to the evidence described above, could or could not have resulted in an intervention effect. The fact that participants in all intervention arms also received regular sessions in groups may have served as an equalizer, but it cannot explain the lack of an overall intervention effect. A possible reason for the lack of an effect may be that, despite the exclusion criterion “already meeting the WHO recommendations for one year preceding baseline,” potential participants may have underreported MVPA to be able to participate in the study. This may have led to higher baseline PA levels than intended and feelings of frustration with intervention messages and materials targeting primarily inactive adults and possibly dropping out of the study. It is also conceivable that potential participants may have overreported PA because of social desirability and may have been excluded, leading to a lack of representation of physically inactive adults in the sample. However, both of these potential explanations are rather speculative. Nevertheless, we conclude that more sensitive strategies are needed to address social desirability concerning the self-reporting of PA during recruitment to better reach initially inactive adults. Owing to the reasons explained above, the results of our study are not generalizable to the general population in this age bracket, and external validity is limited.

Furthermore, previous research has shown that preferences for intervention modality may vary by age, sex, BMI, or social or living environment [15,22,23]. Younger individuals seem to prefer eHealth to print-based interventions [22,23], whereas older or female individuals or those with an adverse weight status appear to be more likely to favor print-based interventions [22]. Unfortunately, in our study, we were not able to investigate variations by sociodemographic characteristics because only 7% of the sample changed groups at follow-up, and preference was not assessed at baseline, but only retrospectively. However, we did not find any group differences in terms of technology readiness. Levels of acceptance were average across groups and competence and control beliefs, as well as willingness to deal with new technologies, were high across groups, suggesting no variations that may have affected decisions for WEB versus PRINT conditions later on in the study.

### Implications for Practice and Research

The great heterogeneity among older adults >60 years (eg, in employment status, chronic disease status, or functional capability) is a key concern and needs to be addressed in future interventions, including differing motivations to participate in the study or to engage in interventions (eg, maintaining functional status). Interventions should include more tailoring in the future, including tailored messages addressing the aspects raised above. One lesson learned in this study was that group sessions paralleling eHealth intervention components contribute to acceptance in this target group and may prevent study dropout. Face-to-face contact with the PA instructor and fellow participants and a sense of structure because of regular weekly meetings were well received by participants in our study. More than two-thirds (131/159, 82.4%) of the participants across groups stated at T1 and T2 that they found the group sessions very or somewhat helpful.

### Conclusions

In conclusion, we successfully adapted and simplified the interventions developed in a previous study. Despite the high acceptance and use of these interventions, no intervention effect was observed for MVPA. Owing to a lack of movement between groups at T1, the role of personal preferences for different delivery modes could not be investigated in full depth.

## Appendix

Multimedia Appendix 1 Baseline characteristics.

Multimedia Appendix 2 World Health Organization recommendations reached.

Multimedia Appendix 3 Adherence, use, and acceptance of the intervention components at the three- and nine-month follow-ups.

Multimedia Appendix 4 CONSORT-eHEALTH checklist (V 1.6.1).

## Abbreviations

cpm: counts per minute
ISCED: International Standard Classification of Education
MMSE-2-BV: Mini-Mental State Examination 2–brief version
MVPA: moderate to vigorous physical activity
PA: physical activity
SB: sedentary behavior
T0: baseline assessment
T1: 3-month follow-up
T2: 9-month follow-up
WHO: World Health Organization
