# Supplementary material for: Web-Based Versus Print-Based Physical Activity Intervention for Community-Dwelling Older Adults: Crossover Randomized Trial
Source: JMIR Mhealth Uhealth. 2022 Mar 23;10(3):e32212. doi: 10.2196/32212 (PMC8987962; doi:10.2196/32212)
Supplement: Multimedia Appendix 1 [file mhealth_v10i3e32212_app1.docx]

**Multimedia Appendix 1. Table S1.** Baseline characteristics.

| Characteristics | | PRINT (n=90) | Web (n=78) | Web+ (n=36) | Total (n=204) |
| --- | --- | --- | --- | --- | --- |
| Age(years), mean (SD); range | | 67.6 (4.9); 60-80 | 67.9 (5.3); 60-80 | 70.5 (6.0); 62-82 | 68.7 (5.4); 60-82 |
| **Sex, n (%)** | | | | | |
|  | Male | 22 (24) | 28 (36) | 19 (53) | 69 (33.8) |
|  | Female | 68 (76) | 50 (64) | 17 (47) | 135 (66.2) |
| **BMI (WHO^a^), n (%)** | | | | | |
|  | Underweight or normal weight | 44 (49) | 28 (36) | 15 (42) | 87 (42.6) |
|  | Overweight/obese | 46 (51) | 50 (64) | 21 (58) | 117 (57.4) |
| **Level of education (ISCED^b^), n (%)** | | | | | |
|  | Low/medium | 37 (41) | 35 (45) | 20 (56) | 92 (45.1) |
|  | High | 53 (59) | 43 (55) | 16 (44) | 112 (54.9) |
| **Family status, n (%)** | | | | | |
|  | Married | 51 (57) | 35 (45) | 24 (67) | 110 (53.9) |
|  | Divorced or single or widowed | 39 (43) | 43 (55) | 12 (33) | 94 (64.1) |
| **Employment status, n (%)** | | | | | |
|  | Employed | 27 (30) | 19 (24) | 5 (14) | 51 (25.0) |
|  | Retired but working | 13 (14) | 16 (11) | 4 (11) | 33 (16.2) |
|  | Retired only | 50 (56) | 43 (55) | 27 (75) | 120 (58.8) |
| **Household income, n (%)** | | | | | |
|  | Low | 24 (27) | 27 (35) | 8 (22) | 59 (28.9) |
|  | Middle | 34 (38) | 28 (36) | 15 (42) | 77 (37.7) |
|  | High | 32 (36) | 23 (30) | 13 (36) | 68 (33.3) |
| **Subjective health status, n (%)** | | | | | |
|  | Excellent or very good | 14 (16) | 10 (13) | 6 (17) | 30 (14.7) |
|  | Good | 62 (69) | 52 (67) | 22 (61) | 136 (66.7) |
|  | Less good or poor | 14 (16) | 16 (21) | 8 (22) | 38 (18.6) |
| **WHO MVPA^c^ recommendation^d^, n (%)** | | | | | |
|  | Not achieved | 75 (83) | 60 (77) | 27 (75) | 162 (79.4) |
|  | Achieved | 11 (12) | 18 (23.1) | 9 (25) | 38 (18.6) |
|  | Missing | 4 (4) | — | — | 4 (2.0) |
| Built environment^e^, mean (SD) | | 3.9 (1) | 3.9 (1) | 4.0 (1) | 3.9 (0.6) |
| Activity-related support^f^, mean (SD) | | 1.6 (1) | 1.7 (1) | 1.8 (1) | 1.7 (0.5) |
| **Ownership and frequency of use of digital devices, mean (SD)** | | | | | |
|  | Number of owned devices^g^ used daily | 1.89 (0.8) | 1.86 (0.8) | 2.03 (0.9) | 1.9 (0.8) |
|  | Frequency of use^i^ (sum of all devices) | 8.95 (2.7) | 8.89 (2.6) | 9.63 (2.9) | 9.0 (2.7) |
| **Technology readiness^h^, mean (SD)** | | | | |  |
|  | Technology acceptance | 2.6 (0.8) | 2.7 (0.8) | 2.9 (0.9) | 2.7 (0.8) |
|  | Technology competence belief | 3.9 (0.8) | 3.9 (0.8) | 3.9 (0.9) | 3.9 (0.8) |
|  | Technology control belief | 3.9 (0.7) | 3.7 (0.7) | 3.9 (0.9) | 3.8 (0.8) |
|  | Technology willingness/readiness | 3.5 (0.6) | 3.4 (0.6) | 3.6 (0.7) | 3.5 (0.6) |
| **Wear time** | | n=86 | n=78 | n=36 | N=200 |
|  | Minutes per day, mean (SD); range | 835.9 (72.8); 624-998 | 826.5 (96.9); 570-1059 | 826.1 (66.8); 711-960 | 830.5 (81.9); 570-1059 |
| **Season, n (%)** | | | | | |
|  | Fall/winter | 41 (46) | 34 (44) | 27 (75) | 102 (50.0) |
|  | Spring/summer | 49 (54) | 44 (56) | 9 (25) | 102 (50.0) |

^a^WHO: World Health Organization.

^b^ISCED: International Standard Classification of Education.

^c^MVPA: moderate to vigorous physical activity.

^d^150 minutes per week.

^e^Scores ranging from 1 to 5, with higher scores indicating higher agreement.

^f^Scores ranging from 1 to 4 with higher scores indicating greater support.

^g^Including computer/notebook, tablet, smartphone, smartwatch, scores ranging from 1 to 5 with *1* indicating daily use and *5* indicating no use or ownership.

^h^Including computer/notebook, tablet, smartphone, and smartwatch, scores ranging from 1 to 16, with higher scores indicating higher frequencies.
