# Supplementary material for: Web-Based Versus Print-Based Physical Activity Intervention for Community-Dwelling Older Adults: Crossover Randomized Trial
Source: JMIR Mhealth Uhealth. 2022 Mar 23;10(3):e32212. doi: 10.2196/32212 (PMC8987962; doi:10.2196/32212)
Supplement: Multimedia Appendix 2 [file mhealth_v10i3e32212_app2.docx]

**Multimedia Appendix 2. Table S2.** WHO MVPA recommendations* according to the analyzed sample

|  | **T0 (N=204)** | | | **T1 (N=159)** | | | **T2 (N=138)** | | |
| --- | --- | --- | --- | --- | --- | --- | --- | --- | --- |
|  | **PRINT (n=90)** | **WEB (n=78)** | **WEB+ (n=36)** | **PRINT (n=73)** | **WEB (n=60)** | **WEB+ (n=26)** | **PRINT (n=67)** | **WEB (n=50)** | **WEB+ (n=21)** |
| Not achieved | 75 (83.3) | 60 (76.9) | 27 (75.0) | 54 (74) | 39 (65) | 18 (69.2) | 47 (70.1) | 33 (66) | 3 (14.3) |
| Achieved | 11 (12.2) | 18 (23.1) | 9 (25.0) | 7 (9.6) | 13 (21.7) | 6 (23.1) | 8 (11.9) | 5(10) | 15 (71.4) |
| Missing | 4 (4.4) |  |  | 12 (16.4) | 8 (13.3) | 2 (7.7) | 12 (17.9) | 12 (24) | 3(14.3) |

***Note:*** *150min/week
