# Supplementary material for: Web-Based Versus Print-Based Physical Activity Intervention for Community-Dwelling Older Adults: Crossover Randomized Trial
Source: JMIR Mhealth Uhealth. 2022 Mar 23;10(3):e32212. doi: 10.2196/32212 (PMC8987962; doi:10.2196/32212)
Supplement: Multimedia Appendix 3 [file mhealth_v10i3e32212_app3.docx]

**Multimedia Appendix 3. Table S3.** Adherence, use and acceptance of the intervention components at the three- and nine-month follow-ups.

|  | **T1** | | |  | **T2** | | |
| --- | --- | --- | --- | --- | --- | --- | --- |
|  | **Print**  n = 73 | **WEB**  n = 60 | **WEB+**  n = 26 |  | **Print**  n = 55 | **WEB**  n = 47 | **WEB+**  n = 36 |
| **Perception that PA recommendations were followed (n, %)** |  |  |  |  |  |  |  |
| Daily | 20 (27.4) | 7 (11.7) | 5 (19.2) |  | 10 (18.2) | 5 (10.6) | 5 (13.9) |
| At least once a week | 31 (42.5) | 25 (41.7) | 7 (26.9) |  | 20 (36.4) | 10 (21.3) | 8 (22.2) |
| Less than once a week | 18 (24.7) | 22 (36.7) | 9 (34.6) |  | 22 (40) | 23 (48.9) | 14 (38.9) |
| Never | 3 (4.1) | 4 (6.7) | 4 (15.4) |  | 2 (3.6) | 5 (10.6) | 6 (16.7) |
| Missing | 1 (1.4) | 2 (3.3) | 1 (3.8) |  | 1 (1.8) | 4 (8.5) | 3 (8.3) |
| **Used the PA diary (n, %)** |  |  |  |  |  |  |  |
| Daily | 25 (34.2) | 18 (30.0) | 4 (15.4) |  | 7 (12.7) | 4 (8.5) | 5 (13.9) |
| At least once a week | 28 (38.4) | 29 (48.3) | 13 (50.0) |  | 17 (30.9) | 19 (40.4) | 16 (44.4) |
| Less than once a week | 16 (21.9) | 6 (10.0) | 6 (23.1) |  | 25 (45.5) | 17 (36.2) | 8 (22.2) |
| Never | 3 (4.1) | 5 (8.3) | 3 (11.5) |  | 4 (7.3) | 2 (4.3) | 3 (8.3) |
| Missing | 1 (1.4) | 2 (3.3) | 0 (0) |  | 2 (3.6) | 5 (10.6) | 4 (11.1) |
| **Used the exercise brochure (n, %)** |  |  |  |  |  |  |  |
| Daily | 19 (26.0) | 9 (15.0) | 3 (11.5) |  | 5 (9.1) | 4 (8.5) | 2 (5.6) |
| At least once a week | 31 (42.5) | 20 (33.3) | 7 (26.9) |  | 17 (30.9) | 10 (21.3) | 8 (22.2) |
| Less than once a week | 19 (26.0) | 24 (40.0) | 11 (42.3) |  | 24 (43.6) | 22 (46.8) | 13 (36.1) |
| Never | 2 (2.7) | 6 (10.0) | 5 (19.2) |  | 6 (10.9) | 6 (12.8) | 10 (27.8) |
| Missing | 2 (2.7) | 1 (1.7) | 0 (0) |  | 3 (5.5) | 5 (10.6) | 3 (8.3) |
| **Used the smartphone app (n, %)** |  |  |  |  |  |  |  |
| Daily |  | 5 (8.3) | 5 (19.2) |  |  | 0 (0) | 6 (16.7) |
| At least once a week |  | 4 (6.7) | 5 (19.2) |  |  | 2 (4.3) | 7 (19.4) |
| Less than once a week |  | 4 (6.7) | 0 (0) |  |  | 4 (8.5) | 4 (11.1) |
| Never |  | 45 (75) | 16 (61.5) |  |  | 33 (70.2) | 15 (41.7) |
| Missing |  | 2 (3.3) | 0 (0) |  |  | 8 (17.0) | 4 (11.1) |
| **Found the program helpful for being physically active (n, %)** |  |  |  |  |  |  |  |
| Rather or very helpful | 46 (63.0) | 46 (76.7) | 19 (73.1) |  | 35 (63.6) | 30 (63.8) | 26 (72.2) |
| Somewhat helpful | 19 (26.0) | 12 (20.0) | 5 (19.2) |  | 14 (25.5) | 13 (27.7) | 7 (19.4) |
| Less helpful or not helpful | 8 (11.0) | 2 (3.3) | 2 (7.7) |  | 4 (7.3) | 2 (4.3) | 0 (0) |
| Missing | 0 (0) | 0 (0) | 0 (0) |  | 2 (3.6) | 2 (4.3) | 3 (8.3) |
| **Found the exercise brochure helpful (n, %)** |  |  |  |  |  |  |  |
| Rather or very helpful | 52 (71.2) | 39 (65) | 16 (61.5) |  | 38 (69.1) | 25 (53.2) | 18 (50) |
| Somewhat helpful | 12 (16.4) | 11 (18.3) | 5 (19.2) |  | 10 (18.2) | 8 (17.0) | 6 (16.7) |
| Less helpful or not helpful | 7 (9.6) | 6 (10.0) | 4 (15.4) |  | 5 (9.1) | 7 (14.9) | 6 (16.7) |
| Missing | 2 (2.7) | 4 (6.7) | 1 (3.8) |  | 2 (3.6) | 7 (14.9) | 6 (16.7) |
| **Found the printed material/website helpful (n, %)** |  |  |  |  |  |  |  |
| Rather or very helpful | 42 (57.5) | 41 (68.3) | 16 (61.5) |  | 34 (61.8) | 27 (57.4) | 22 (61.1) |
| Somewhat helpful | 24 (32.9) | 11 (18.3) | 7 (26.9) |  | 11 (20) | 13 (27.7) | 6 (16.7) |
| Less helpful or not helpful | 6 (8.2) | 2 (3.3) | 3 (11.5) |  | 7 (12.7) | 1 (2.1) | 3 (8.3) |
| Missing | 1 (1.4) | 6 (10.0) | 0 (0) |  | 3 (5.5) | 6 (12.8) | 5 (13.9) |
| **Found the smartphone app helpful (n, %)** |  |  |  |  |  |  |  |
| Rather or very helpful |  | 12 (20.0) | 10 (38.5) |  |  | 3 (6.4) | 13 (36.1) |
| Somewhat helpful |  | 4 (6.7) | 1 (3.8) |  |  | 3 (6.4) | 3 (8.3) |
| Less helpful or not helpful |  | 30 (50.0) | 13 (50.0) |  |  | 34 (72.3) | 13 (36.1) |
| Missing |  | 14 (23.3) | 2 (7.7) |  |  | 7 (14.9) | 7 (19.4) |
| **Found the PA tracker helpful (n, %)** |  |  |  |  |  |  |  |
| Rather or very helpful |  |  | 20 (76.9) |  |  |  | 26 (72.2) |
| Somewhat helpful |  |  | 2 (7.7) |  |  |  | 3 (8.3) |
| Less helpful or not helpful |  |  | 4 (15.4) |  |  |  | 3 (8.3) |
| Missing |  |  | 0 (0) |  |  |  | 4 (11.1) |
| **Found the group sessions helpful (n, %)** |  |  |  |  |  |  |  |
| Rather or very helpful | 54 (74.0) | 41 (68.3) | 19 (73.1) |  | 35 (63.6) | 22 (46.8) | 23 (63.9) |
| Somewhat helpful | 8 (11.0) | 7 (11.7) | 2 (7.7) |  | 5 (9.1) | 7 (14.9) | 2 (5.6) |
| Less helpful or not helpful | 6 (8.2) | 6 (10.0) | 1 (3.8) |  | 1 (1.8) | 3 (6.4) | 4 (11.1) |
| Missing | 5 (6.8) | 6 (10.0) | 4 (15.4) |  | 14 (25.5) | 15 (31.9) | 7 (19.4) |
| **Would recommend the program to others (n, %)** |  |  |  |  |  |  |  |
| Probably or definitely | 48 (65.8) | 46 (76.7) | 18 (69.2) |  | 42 (76.4) | 30 (63.8) | 31 (86.1) |
| Maybe | 15 (20.5) | 11 (18.3) | 6 (23.1) |  | 6 (10.9) | 11 (23.4) | 3 (8.3) |
| Rather not or definitely not | 10 (13.7) | 2 (3.3) | 2 (7.7) |  | 5 (9.1) | 3 (6.4) | 1 (2.8) |
| Missing | 0 (0) | 1 (1.7) | 0 (0) |  | 0 (0) | 3 (6.4) | 1 (2.8) |
| **Preference-matched randomization** |  |  |  |  | 49 (73.1) | 39 (78.0) | 16 (76.2) |
| **Mean number of attended weekly/monthly group sessions** (M, SD) | 8.0 (2.0) | 7.8 (2.3) | 8.2 (1.9) |  | 2.4 (0.7) | 2.3 (0.8) | 2.2 (0.8) |

***Note:*** The analyzed sample (n in the top row) corresponds with the number of participants who answered the follow-up questionnaires. Group membership at T2 represents the group membership after cross-over. PA: physical activity.
