# Supplementary material for: Web-Based Versus Print-Based Physical Activity Intervention for Community-Dwelling Older Adults: Crossover Randomized Trial
Source: JMIR Mhealth Uhealth. 2022 Mar 23;10(3):e32212. doi: 10.2196/32212 (PMC8987962; doi:10.2196/32212)
Supplement: Multimedia Appendix 4 [file mhealth_v10i3e32212_app4.pdf]

CONSORT-EHEALTH (V 1.6.1) - Submission/Publication Form

The CONSORT-EHEALTH checklist is intended for authors of randomized trials evaluating web-based and Internet-based applications/interventions, including mobile interventions, electronic games (incl multiplayer games), social media, certain telehealth applications, and other interactive and/or networked electronic applications. Some of the items (e.g. all subitems under item 5 - description of the intervention) may also be applicable for other study designs.

The goal of the CONSORT EHEALTH checklist and guideline is to be
a) a guide for reporting for authors of RCTs,
b) to form a basis for appraisal of an ehealth trial (in terms of validity)

CONSORT-EHEALTH items/subitems are MANDATORY reporting items for studies published in the Journal of Medical Internet Research and other journals / scientific societies endorsing the checklist.

Items numbered 1., 2., 3., 4a., 4b etc are original CONSORT or CONSORT-NPT (non-pharmacologic treatment) items.
Items with Roman numerals (i., ii, iii, iv etc.) are CONSORT-EHEALTH extensions/clarifications.

As the CONSORT-EHEALTH checklist is still considered in a formative stage, we would ask that you also RATE ON A SCALE OF 1-5 how important/useful you feel each item is FOR THE PURPOSE OF THE CHECKLIST and reporting guideline (optional).

Mandatory reporting items are marked with a red *.
In the textboxes, either copy & paste the relevant sections from your manuscript into this form - please include any quotes from your manuscript in QUOTATION MARKS,
or answer directly by providing additional information not in the manuscript, or elaborating on why the item was not relevant for this study.

YOUR ANSWERS WILL BE PUBLISHED AS A SUPPLEMENTARY FILE TO YOUR PUBLICATION IN JMIR AND ARE CONSIDERED PART OF YOUR PUBLICATION (IF ACCEPTED).
Please fill in these questions diligently. Information will not be copyedited, so please use proper spelling and grammar, use correct capitalization, and avoid abbreviations.

DO NOT FORGET TO SAVE AS PDF _AND_ CLICK THE SUBMIT BUTTON SO YOUR ANSWERS ARE IN OUR DATABASE !!!

Citation Suggestion (if you append the pdf as Appendix we suggest to cite this paper in the caption):
Eysenbach G, CONSORT-EHEALTH Group
CONSORT-EHEALTH: Improving and Standardizing Evaluation Reports of Web-based and Mobile Health Interventions
J Med Internet Res 2011;13(4):e126
URL: http://www.jmir.org/2011/4/e126/

doi: 10.2196/jmir.1923
PMID: 22209829

* Erforderlich

Your name *

First Last

Claudia Pischke

Primary Affiliation (short), City, Country *

University of Toronto, Toronto, Canada

Heinrich Heine University Düsseldorf

Your e-mail address *

abc@gmail.com

ClaudiaRuth.Pischke@med.uni-duesseldorf.de

Title of your manuscript *

Provide the (draft) title of your manuscript.

A Web-based versus a Print-based Physical Activity Intervention for Community-dwelling Older Adults: Randomized Trial With a Cross-over Design

Name of your App/Software/Intervention *

If there is a short and a long/alternate name, write the short name first and add the long name in brackets.

Fit im Nordwesten

Evaluated Version (if any)

e.g. "V1", "Release 2017-03-01", "Version 2.0.27913"

not applicable

Language(s) *

What language is the intervention/app in? If multiple languages are available, separate by comma (e.g. "English, French")

German

URL of your Intervention Website or App

e.g. a direct link to the mobile app on app in appstore (itunes, Google Play), or URL of the website. If the intervention is a DVD or hardware, you can also link to an Amazon page.

http://doi.org/10.5281/zenodo.4568235

URL of an image/screenshot (optional)

Meine Antwort

(!) Geben Sie eine gültige URL ein.

Accessibility *

Can an enduser access the intervention presently?

- ○ access is free and open
- ◉ access only for special usergroups, not open
- ○ access is open to everyone, but requires payment/subscription/in-app purchases
- ○ app/intervention no longer accessible
- ○ Sonstiges:

Primary Medical Indication/Disease/Condition *

e.g. "Stress", "Diabetes", or define the target group in brackets after the condition, e.g. "Autism (Parents of children with)", "Alzheimers (Informal Caregivers of)"

not applicable (primary prevention targeting ol

Primary Outcomes measured in trial *

comma-separated list of primary outcomes reported in the trial

Physical activity

Secondary/other outcomes

Are there any other outcomes the intervention is expected to affect?

It is also expected to affect sedentary behavior.

Recommended "Dose" *

What do the instructions for users say on how often the app should be used?

- ○ Approximately Daily
- ◉ Approximately Weekly
- ○ Approximately Monthly
- ○ Approximately Yearly
- ○ "as needed"
- ○ Sonstiges:

Approx. Percentage of Users (starters) still using the app as recommended after 3 months *

- (●) unknown / not evaluated
- ( ) 0-10%
- ( ) 11-20%
- ( ) 21-30%
- ( ) 31-40%
- ( ) 41-50%
- ( ) 51-60%
- ( ) 61-70%
- ( ) 71%-80%
- ( ) 81-90%
- ( ) 91-100%
- ( ) Sonstiges:

Overall, was the app/intervention effective? *

- ( ) yes: all primary outcomes were significantly better in intervention group vs control
- ( ) partly: SOME primary outcomes were significantly better in intervention group vs control
- ( ) no statistically significant difference between control and intervention
- ( ) potentially harmful: control was significantly better than intervention in one or more outcomes
- (●) inconclusive: more research is needed
- ( ) Sonstiges:

Article Preparation Status/Stage *

At which stage in your article preparation are you currently (at the time you fill in this form)

- ( ) not submitted yet - in early draft status
- ( ) not submitted yet - in late draft status, just before submission
- (•) submitted to a journal but not reviewed yet
- ( ) submitted to a journal and after receiving initial reviewer comments
- ( ) submitted to a journal and accepted, but not published yet
- ( ) published
- ( ) Sonstiges:

Journal *

If you already know where you will submit this paper (or if it is already submitted), please provide the journal name (if it is not JMIR, provide the journal name under "other")

- ( ) not submitted yet / unclear where I will submit this
- ( ) Journal of Medical Internet Research (JMIR)
- (•) JMIR mHealth and UHealth
- ( ) JMIR Serious Games
- ( ) JMIR Mental Health
- ( ) JMIR Public Health
- ( ) JMIR Formative Research
- ( ) Other JMIR sister journal
- ( ) Sonstiges:

Is this a full powered effectiveness trial or a pilot/feasibility trial? *

- ○ Pilot/feasibility
- ◉ Fully powered

Manuscript tracking number *

If this is a JMIR submission, please provide the manuscript tracking number under "other" (The ms tracking number can be found in the submission acknowledgement email, or when you login as author in JMIR. If the paper is already published in JMIR, then the ms tracking number is the four-digit number at the end of the DOI, to be found at the bottom of each published article in JMIR)

- ○ no ms number (yet) / not (yet) submitted to / published in JMIR
- ◉ Sonstiges: 32212-524289-1-SM

TITLE AND ABSTRACT

1a) TITLE: Identification as a randomized trial in the title

1a) Does your paper address CONSORT item 1a? *

I.e does the title contain the phrase "Randomized Controlled Trial"? (if not, explain the reason under "other")

- ○ yes
- ◉ Sonstiges: It is a randomized trial with a crossover design which is mentione

1a-i) Identify the mode of delivery in the title

Identify the mode of delivery. Preferably use "web-based" and/or "mobile" and/or "electronic game" in the title. Avoid ambiguous terms like "online", "virtual", "interactive". Use "Internet-based" only if Intervention includes non-web-based Internet components (e.g. email), use "computer-based" or "electronic" only if offline products are used. Use "virtual" only in the context of "virtual reality" (3-D worlds). Use "online" only in the context of "online support groups". Complement or substitute product names with broader terms for the class of products (such as "mobile" or "smart phone" instead of "iphone"), especially if the application runs on different platforms.

	1	2	3	4	5	
subitem not at all important	○	○	○	○	◉	essential

Auswahl löschen

Does your paper address subitem 1a-i? *

Copy and paste relevant sections from manuscript title (include quotes in quotation marks "like this" to indicate direct quotes from your manuscript), or elaborate on this item by providing additional information not in the ms, or briefly explain why the item is not applicable/relevant for your study

"A Web-based versus a Print-based Physical Activity Intervention for Community-dwelling Older Adults: Randomized Trial With a Cross-over Design"

1a-ii) Non-web-based components or important co-interventions in title

Mention non-web-based components or important co-interventions in title, if any (e.g., "with telephone support").

	1	2	3	4	5	
subitem not at all important	○	○	○	○	◉	essential

Auswahl löschen

Does your paper address subitem 1a-ii?

Copy and paste relevant sections from manuscript title (include quotes in quotation marks "like this" to indicate direct quotes from your manuscript), or elaborate on this item by providing additional information not in the ms, or briefly explain why the item is not applicable/relevant for your study

"A Web-based versus a Print-based Physical Activity Intervention for Community-dwelling Older Adults: Randomized Trial With a Cross-over Design"

1a-iii) Primary condition or target group in the title

Mention primary condition or target group in the title, if any (e.g., "for children with Type I Diabetes")
Example: A Web-based and Mobile Intervention with Telephone Support for Children with Type I Diabetes: Randomized Controlled Trial

	1	2	3	4	5	
subitem not at all important	○	○	○	○	◉	essential

Auswahl löschen

Does your paper address subitem 1a-iii? *

Copy and paste relevant sections from manuscript title (include quotes in quotation marks "like this" to indicate direct quotes from your manuscript), or elaborate on this item by providing additional information not in the ms, or briefly explain why the item is not applicable/relevant for your study

"Community-dwelling Older Adults"

1b) ABSTRACT: Structured summary of trial design, methods, results, and conclusions

NPT extension: Description of experimental treatment, comparator, care providers, centers, and blinding status.

1b-i) Key features/functionalities/components of the intervention and comparator in the METHODS section of the ABSTRACT

Mention key features/functionalities/components of the intervention and comparator in the abstract. If possible, also mention theories and principles used for designing the site. Keep in mind the needs of systematic reviewers and indexers by including important synonyms. (Note: Only report in the abstract what the main paper is reporting. If this information is missing from the main body of text, consider adding it)

	1	2	3	4	5	
subitem not at all important	○	○	○	○	◉	essential

Auswahl löschen

Does your paper address subitem 1b-i? *

Copy and paste relevant sections from the manuscript abstract (include quotes in quotation marks "like this" to indicate direct quotes from your manuscript), or elaborate on this item by providing additional information not in the ms, or briefly explain why the item is not applicable/relevant for your study

"Participants were recruited offline and randomized to one of the following interventions for self-monitoring PA: (a) a print-based intervention (PRINT n=113), (b) a web-based intervention (WEB, n=129). Thirty percent (n=38) of those in group (b) received a PA tracker in addition to WEB (WEB+, (c))."

1b-ii) Level of human involvement in the METHODS section of the ABSTRACT

Clarify the level of human involvement in the abstract, e.g., use phrases like "fully automated" vs. "therapist/nurse/care provider/physician-assisted" (mention number and expertise of providers involved, if any). (Note: Only report in the abstract what the main paper is reporting. If this information is missing from the main body of text, consider adding it)

	1	2	3	4	5	
subitem not at all important	○	○	○	○	◉	essential

Auswahl löschen

Does your paper address subitem 1b-ii?

Copy and paste relevant sections from the manuscript abstract (include quotes in quotation marks "like this" to indicate direct quotes from your manuscript), or elaborate on this item by providing additional information not in the ms, or briefly explain why the item is not applicable/relevant for your study

"All intervention groups were offered ten weekly face-to-face group sessions led by trained assistants. Afterwards, participants could choose to stay in their group or cross over to one of the other groups and group sessions were continued monthly for another six months."

1b-iii) Open vs. closed, web-based (self-assessment) vs. face-to-face assessments in the METHODS section of the ABSTRACT

Mention how participants were recruited (online vs. offline), e.g., from an open access website or from a clinic or a closed online user group (closed usergroup trial), and clarify if this was a purely web-based trial, or there were face-to-face components (as part of the intervention or for assessment). Clearly say if outcomes were self-assessed through questionnaires (as common in web-based trials). Note: In traditional offline trials, an open trial (open-label trial) is a type of clinical trial in which both the researchers and participants know which treatment is being administered. To avoid confusion, use "blinded" or "unblinded" to indicated the level of blinding instead of "open", as "open" in web-based trials usually refers to "open access" (i.e. participants can self-enrol). (Note: Only report in the abstract what the main paper is reporting. If this information is missing from the main body of text, consider adding it)

	1	2	3	4	5	
subitem not at all important	○	○	○	○	◉	essential

Auswahl löschen

Does your paper address subitem 1b-iii?

Copy and paste relevant sections from the manuscript abstract (include quotes in quotation marks "like this" to indicate direct quotes from your manuscript), or elaborate on this item by providing additional information not in the ms, or briefly explain why the item is not applicable/relevant for your study

"Participants were recruited offline and randomized to one of the following interventions for self-monitoring PA: (a) a print-based intervention (PRINT n=113), (b) a web-based intervention (WEB, n=129). Thirty percent (n=38) of those in group (b) received a PA tracker in addition to WEB (WEB+, (c)). After randomization, participants and researchers were not blinded."

1b-iv) RESULTS section in abstract must contain use data

Report number of participants enrolled/assessed in each group, the use/uptake of the intervention (e.g., attrition/adherence metrics, use over time, number of logins etc.), in addition to primary/secondary outcomes. (Note: Only report in the abstract what the main paper is reporting. If this information is missing from the main body of text, consider adding it)

	1	2	3	4	5	
subitem not at all important	○	○	○	○	◉	essential

Auswahl löschen

Does your paper address subitem 1b-iv?

Copy and paste relevant sections from the manuscript abstract (include quotes in quotation marks "like this" to indicate direct quotes from your manuscript), or elaborate on this item by providing additional information not in the ms, or briefly explain why the item is not applicable/relevant for your study

"One-hundred and ninety-five participants completed T1. Only n=1 changed from WEB to PRINT and n=15 moved from PRINT to WEB (WEB-WEB: n=103, PRINT-PRINT: n=76) when offered to cross over at T1. One-hundred and sixty participants completed T2."

1b-v) CONCLUSIONS/DISCUSSION in abstract for negative trials

Conclusions/Discussions in abstract for negative trials: Discuss the primary outcome - if the trial is negative (primary outcome not changed), and the intervention was not used, discuss whether negative results are attributable to lack of uptake and discuss reasons. (Note: Only report in the abstract what the main paper is reporting. If this information is missing from the main body of text, consider adding it)

	1	2	3	4	5	
subitem not at all important	○	○	○	○	◉	essential

Auswahl löschen

Does your paper address subitem 1b-v?

Copy and paste relevant sections from the manuscript abstract (include quotes in quotation marks "like this" to indicate direct quotes from your manuscript), or elaborate on this item by providing additional information not in the ms, or briefly explain why the item is not applicable/relevant for your study

"Despite high levels of acceptance of web- and print-based interventions for PA promotion and little movement between groups at T1, when given the choice, participation was not associated with increases in PA or decreases in SB over time."

INTRODUCTION

2a) In INTRODUCTION: Scientific background and explanation of rationale

2a-i) Problem and the type of system/solution

Describe the problem and the type of system/solution that is object of the study: intended as stand-alone intervention vs. incorporated in broader health care program? Intended for a particular patient population? Goals of the intervention, e.g., being more cost-effective to other interventions, replace or complement other solutions? (Note: Details about the intervention are provided in "Methods" under 5)

	1	2	3	4	5	
subitem not at all important	○	○	○	○	◉	essential

Auswahl löschen

Does your paper address subitem 2a-i? *

Copy and paste relevant sections from the manuscript (include quotes in quotation marks "like this" to indicate direct quotes from your manuscript), or elaborate on this item by providing additional information not in the ms, or briefly explain why the item is not applicable/relevant for your study

"Depending on group assignment, all intervention material was either provided as printed material or made available on the website. The smartphone application (app) additionally provided access to the exercises and the PA diary for individuals in WEB and WEB+. On the website, in the android web-app, as well as in the printed diary, weekly feedback regarding whether PA goals were reached was provided (see figure 2 in (31)) The number of minutes or units exercised were displayed, as well as units required in order to reach the goal. The WEB+ group also used a PA tracker (Fitbit Zip, Fitbit Inc., San Francisco) in addition to the website or app, and the daily step count tracked with the device was synchronized with the website. No prompts or reminders were used on the website or in the app."

2a-ii) Scientific background, rationale: What is known about the (type of) system

Scientific background, rationale: What is known about the (type of) system that is the object of the study (be sure to discuss the use of similar systems for other conditions/diagnoses, if appropiate), motivation for the study, i.e. what are the reasons for and what is the context for this specific study, from which stakeholder viewpoint is the study performed, potential impact of findings [2]. Briefly justify the choice of the comparator.

	1	2	3	4	5	
subitem not at all important	○	○	○	○	◉	essential

Auswahl löschen

Does your paper address subitem 2a-ii? *

Copy and paste relevant sections from the manuscript (include quotes in quotation marks "like this" to indicate direct quotes from your manuscript), or elaborate on this item by providing additional information not in the ms, or briefly explain why the item is not applicable/relevant for your study

"Less than half of the German adults aged 65 years and above meet the (former) recommendations for endurance training (42%) and only one third meet the strength training recommendations [6]. However, compared to the EU average of adults in this age segment, German men and women display slightly higher proportions of adults reaching the recommendations [8]. Further, results based on the European Health Interview Survey (EHIS) and the Survey of Health Ageing and Retirement in Europe (SHARE) looking at associations between the proportion of European adults above the age of 65 years reaching the recommendation of >150 min of PA per week and the proportion of prefrail or frail individuals suggest a negative association [9]. To prevent frailty in older adults, Haider and colleagues call for "community-based approaches aimed at achieving PA recommendations" (p.292, [9]) at the population-level and the creation of built environments enabling PA [9]. Previous research conducted in Germany indicates that population-based approaches to increase PA, such as mass media campaigns, community-based multi-component interventions, and environmental approaches can be effective in the general population [10]. In addition, individual-level interventions provide opportunities to further increase the effect of such population-level approaches [1, 11-13]. However, the role of different modalities for delivering these intervention approaches to older adults remains unclear.
Results of several systematic reviews indicate that participation in interventions providing information on PA face-to-face or via printed materials leads to increased PA levels in older adults [14-16]. Engagement in (interactive) web-based PA interventions is also associated with increased MVPA, walking, and a higher daily step count in intervention vs. control groups [17-18]. Further, results of a systematic review evaluating the effectiveness of eHealth compared to non-eHealth or no intervention in adults aged 55 years and above suggest that eHealth interventions can effectively promote PA in the short-term [13], but there is still a lack of evidence regarding long-term effects. Recent evidence from a review of reviews examining effects and characteristics of PA promotion interventions aimed at community-dwelling adults over 50 years of age indicates that increases in PA can be sustained for up to twelve months [19]. To conclude, it is still unclear whether eHealth interventions have a greater impact on PA behavior than non-eHealth (e.g., print-based) interventions in adults aged 65 years and older and whether increased levels of PA can be maintained over longer periods of time.
Further, the influence of individual preferences for intervention modality and variances in the impact on intervention outcomes is still not well understood [20, 21]. Previous studies suggest that preferences may vary by age, gender, BMI or by social or living environment [22-24]. For example, preference for a web-based intervention was positively related to younger age [23-24] and to high internet use and was negatively associated with female gender. Conversely, older, female and obese individuals were more likely to choose a print-based intervention [23]. These variations by socio-demographic characteristics may also explain differences in the use of PA trackers [25]. In order to increase the impact of this already shown to be effective tool [26], the use of trackers in PA interventions should be aligned with preferences of different target groups [22]. Both, retention in intervention studies and adherence to intervention components, may improve, if individual preferences for interventions are taken into account [12, 22]. Hence, in this study, a cross-over design was employed to examine the role of personal preferences for different delivery modes with regard to intervention effectiveness.

This study (PROMOTE II) was funded by the Federal Ministry of Education and Research (BMBF), as part of the Physical Activity and Health Equity: Primary Prevention for Healthy Ageing (AEQUIPA) research network [27]. It builds on the results of a previous study embedded in the network (PROMOTE I, [28-30]) which tested the effectiveness of two tailored web-based interventions for the promotion of a physically active lifestyle in adults aged 65 to 75 years in a community-based intervention trial against a delayed-intervention control group. In the previous study, we found relatively high baseline levels of PA in intervention participants. Based on this observation, individuals who had been regularly physically active for at least 2.5 hours per week for more than one year were excluded during recruitment for the current study. Further, study dropout was higher in the group assigned to use PA trackers in addition to a website compared to the website only group indicating that randomization to a modality which was not a preference led to participants deciding to quit the intervention [28-30].
Based on these results gathered in the preceding study, this study included four aims:
a) adapt and simplify the web-based intervention of the previous study to further improve usability and develop a simple print-based intervention that initially inactive participants with little affinity to technology find easy to use;
b) investigate acceptance and use of two interventions (web- vs. print-based), as well as changes in PA, among older adults (aged 60 years and above) in a randomized trial with a cross-over design over the course of nine months;
c) examine the role of personal preferences for different delivery modes with regard to intervention effectiveness;
d) explore associations of changes in PA with possible changes in physical fitness and cognitive capacity in a pooled sample of participants of both PROMOTE I and II trials.
In this article, we report results addressing the first three study aims. Results addressing the last aim will be reported in a subsequent article. We hypothesized that both interventions would significantly increase MVPA and decrease sedentary behavior (SB) at the first and second follow-ups [31]."

2b) In INTRODUCTION: Specific objectives or hypotheses

Does your paper address CONSORT subitem 2b? *

Copy and paste relevant sections from the manuscript (include quotes in quotation marks "like this" to indicate direct quotes from your manuscript), or elaborate on this item by providing additional information not in the ms, or briefly explain why the item is not applicable/relevant for your study

"Based on these results gathered in the preceding study, this study included four aims:
a) adapt and simplify the web-based intervention of the previous study to further improve usability and develop a simple print-based intervention that initially inactive participants with little affinity to technology find easy to use;
b) investigate acceptance and use of two interventions (web- vs. print-based), as well as changes in PA, among older adults (aged 60 years and above) in a randomized trial with a cross-over design over the course of nine months;
c) examine the role of personal preferences for different delivery modes with regard to intervention effectiveness."

METHODS

3a) Description of trial design (such as parallel, factorial) including allocation ratio

Does your paper address CONSORT subitem 3a? *

Copy and paste relevant sections from the manuscript (include quotes in quotation marks "like this" to indicate direct quotes from your manuscript), or elaborate on this item by providing additional information not in the ms, or briefly explain why the item is not applicable/relevant for your study

"Of a total of n=3,660 older adults invited, n=823 individuals were assessed for eligibility during computer-assisted telephone interviews (Figure 1). In total, n=581 potential participants were excluded. After determination of eligibility, n=242 included study participants were randomized to one of two groups by the study nurse applying an allocation ratio of 1 to 1: (a) a print intervention with subjective PA self-monitoring via printed PA-pyramid (PRINT, n=113), (b) a web-based intervention with subjective PA self-monitoring via a web-based PA-pyramid (WEB, n=129). Thirty percent of those in group (b) were randomly selected and received a PA tracker (objective PA self-monitoring) in addition (WEB+, group (c), n=38). Weekly time slots were randomly assigned to the three intervention groups. Participants were blinded to the intervention group during randomization (i.e., they were free to choose from available time slots during the telephone interview with the study nurse without knowing which intervention group they were then assigned to)."

3b) Important changes to methods after trial commencement (such as eligibility criteria), with reasons

Does your paper address CONSORT subitem 3b? *

Copy and paste relevant sections from the manuscript (include quotes in quotation marks "like this" to indicate direct quotes from your manuscript), or elaborate on this item by providing additional information not in the ms, or briefly explain why the item is not applicable/relevant for your study

No changes were made after commencement.

3b-i) Bug fixes, Downtimes, Content Changes

Bug fixes, Downtimes, Content Changes: ehealth systems are often dynamic systems. A description of changes to methods therefore also includes important changes made on the intervention or comparator during the trial (e.g., major bug fixes or changes in the functionality or content) (5-iii) and other "unexpected events" that may have influenced study design such as staff changes, system failures/downtimes, etc. [2].

	1	2	3	4	5	
subitem not at all important	◉	○	○	○	○	essential

Auswahl löschen

Does your paper address subitem 3b-i?

Copy and paste relevant sections from the manuscript (include quotes in quotation marks "like this" to indicate direct quotes from your manuscript), or elaborate on this item by providing additional information not in the ms, or briefly explain why the item is not applicable/relevant for your study

Not applicable, because no bug fixes were performed.

4a) Eligibility criteria for participants

Does your paper address CONSORT subitem 4a? *

Copy and paste relevant sections from the manuscript (include quotes in quotation marks "like this" to indicate direct quotes from your manuscript), or elaborate on this item by providing additional information not in the ms, or briefly explain why the item is not applicable/relevant for your study

"Briefly, individuals were included in the study, if they were aged 60 years and above, lived independently and provided an informed consent. Individuals were excluded from the study if they reported that they had been regularly physically active for at least 2.5 hours per week for more than one year. Further, having participated in the previous trial, a planned vacation during the intervention period exceeding two weeks, a medical condition / diagnosis prohibiting PA, severe visual or other impairments, implanted devices or occasional syncopes led to exclusion (for further detail, see (31)). Cognitive state was measured with the Mini-Mental-State Examination 2 - brief version (MMSE-2-BV) and the exclusion criterion was initially set to a MMSE-2-BV score of ≤14. As the manual for the MMSE-2-BV does not define a cut-off value for determination of cognitive impairment, the initially chosen cut-off was re-evaluated during the study and turned out to be too conservative. Based on previous studies [32,33], the cut-off was therefore adapted and individuals with an MMSE-2-BV score <13 were excluded."

4a-i) Computer / Internet literacy

Computer / Internet literacy is often an implicit "de facto" eligibility criterion - this should be explicitly clarified.

	1	2	3	4	5	
subitem not at all important	◉	○	○	○	○	essential

Auswahl löschen

Does your paper address subitem 4a-i?

Copy and paste relevant sections from the manuscript (include quotes in quotation marks "like this" to indicate direct quotes from your manuscript), or elaborate on this item by providing additional information not in the ms, or briefly explain why the item is not applicable/relevant for your study

Computer literacy was not an implicit exclusion criteria.

4a-ii) Open vs. closed, web-based vs. face-to-face assessments:

Open vs. closed, web-based vs. face-to-face assessments: Mention how participants were recruited (online vs. offline), e.g., from an open access website or from a clinic, and clarify if this was a purely web-based trial, or there were face-to-face components (as part of the intervention or for assessment), i.e., to what degree got the study team to know the participant. In online-only trials, clarify if participants were quasi-anonymous and whether having multiple identities was possible or whether technical or logistical measures (e.g., cookies, email confirmation, phone calls) were used to detect/prevent these.

	1	2	3	4	5	
subitem not at all important	(●)	○	○	○	○	essential

Auswahl löschen

Does your paper address subitem 4a-ii? *

Copy and paste relevant sections from the manuscript (include quotes in quotation marks "like this" to indicate direct quotes from your manuscript), or elaborate on this item by providing additional information not in the ms, or briefly explain why the item is not applicable/relevant for your study

Recruitment was performed offline.

4a-iii) Information giving during recruitment

Information given during recruitment. Specify how participants were briefed for recruitment and in the informed consent procedures (e.g., publish the informed consent documentation as appendix, see also item X26), as this information may have an effect on user self-selection, user expectation and may also bias results.

	1	2	3	4	5	
subitem not at all important	○	○	○	○	(●)	essential

Auswahl löschen

Does your paper address subitem 4a-iii?

Copy and paste relevant sections from the manuscript (include quotes in quotation marks "like this" to indicate direct quotes from your manuscript), or elaborate on this item by providing additional information not in the ms, or briefly explain why the item is not applicable/relevant for your study

"The study obtained ethical approval from the Medical Association Bremen, Germany on July 3rd, 2018. Potential participants were informed about the study during the initial telephone interviews and were fully informed during an introductory face-to-face briefing session. They were also told that they would be randomized to one of the intervention groups and knew about their existence. At the end of the introductory session, all participants were fully informed about the study and provided informed consent. Participants were not blinded to their intervention group neither were any research staff conducting the study or the statistician analyzing the data."

4b) Settings and locations where the data were collected

Does your paper address CONSORT subitem 4b? *

Copy and paste relevant sections from the manuscript (include quotes in quotation marks "like this" to indicate direct quotes from your manuscript), or elaborate on this item by providing additional information not in the ms, or briefly explain why the item is not applicable/relevant for your study

"Two weeks before the intervention started, at the introductory event, participants received the questionnaires for the baseline assessment (T0) and were instructed on how and when to wear the accelerometer to measure their baseline PA behavior (cf. below). They were asked to bring along this data collection material to their first weekly group session, where they underwent a short version of the MMSE-2-BV which was conducted individually in a separate room by research staff [34]. During the first sessions of the three- and nine-months follow-ups (T1 and T2), study participants received the data collection material in person from the research staff and were asked to send it back within one week via mail."

4b-i) Report if outcomes were (self-)assessed through online questionnaires

Clearly report if outcomes were (self-)assessed through online questionnaires (as common in web-based trials) or otherwise.

	1	2	3	4	5	
subitem not at all important	◉	○	○	○	○	essential

Auswahl löschen

Does your paper address subitem 4b-i? *

Copy and paste relevant sections from the manuscript (include quotes in quotation marks "like this" to indicate direct quotes from your manuscript), or elaborate on this item by providing additional information not in the ms, or briefly explain why the item is not applicable/relevant for your study

Assesments were performed offline.

4b-ii) Report how institutional affiliations are displayed

Report how institutional affiliations are displayed to potential participants [on ehealth media], as affiliations with prestigious hospitals or universities may affect volunteer rates, use, and reactions with regards to an intervention.(Not a required item – describe only if this may bias results)

	1	2	3	4	5	
subitem not at all important	◉	○	○	○	○	essential

Auswahl löschen

Does your paper address subitem 4b-ii?

Copy and paste relevant sections from the manuscript (include quotes in quotation marks "like this" to indicate direct quotes from your manuscript), or elaborate on this item by providing additional information not in the ms, or briefly explain why the item is not applicable/relevant for your study

Meine Antwort

5) The interventions for each group with sufficient details to allow replication, including how and when they were actually administered

5-i) Mention names, credential, affiliations of the developers, sponsors, and owners

Mention names, credential, affiliations of the developers, sponsors, and owners [6] (if authors/evaluators are owners or developer of the software, this needs to be declared in a "Conflict of interest" section or mentioned elsewhere in the manuscript).

	1	2	3	4	5	
subitem not at all important	◉	○	○	○	○	essential

Auswahl löschen

Does your paper address subitem 5-i?

Copy and paste relevant sections from the manuscript (include quotes in quotation marks "like this" to indicate direct quotes from your manuscript), or elaborate on this item by providing additional information not in the ms, or briefly explain why the item is not applicable/relevant for your study

Authors are the owners.

(!) Your answer must have a minimum of 25 characters.

5-ii) Describe the history/development process

Describe the history/development process of the application and previous formative evaluations (e.g., focus groups, usability testing), as these will have an impact on adoption/use rates and help with interpreting results.

	1	2	3	4	5	
subitem not at all important	○	○	◉	○	○	essential

Auswahl löschen

Does your paper address subitem 5-ii?

Copy and paste relevant sections from the manuscript (include quotes in quotation marks "like this" to indicate direct quotes from your manuscript), or elaborate on this item by providing additional information not in the ms, or briefly explain why the item is not applicable/relevant for your study

The design process of the interventions and their content are described elsewhere: Pischke CR, Voelcker-Rehage C, Peters M, Ratz T, Pohlabeln H, Meyer J, von Holdt K, Lippke S. Implementation and effects of information technology-based and print-based interventions to promote physical activity among community-dwelling older adults: Protocol for a randomized crossover trial. JMIR Res Protoc. 2020 Apr 27;9(4):e15168. doi: 10.2196/15168.

5-iii) Revisions and updating

Revisions and updating. Clearly mention the date and/or version number of the application/intervention (and comparator, if applicable) evaluated, or describe whether the intervention underwent major changes during the evaluation process, or whether the development and/or content was "frozen" during the trial. Describe dynamic components such as news feeds or changing content which may have an impact on the replicability of the intervention (for unexpected events see item 3b).

	1	2	3	4	5	
subitem not at all important	◉	○	○	○	○	essential

Auswahl löschen

Does your paper address subitem 5-iii?

Copy and paste relevant sections from the manuscript (include quotes in quotation marks "like this" to indicate direct quotes from your manuscript), or elaborate on this item by providing additional information not in the ms, or briefly explain why the item is not applicable/relevant for your study

The versions of the intervention were not changed during the trial.

5-iv) Quality assurance methods

Provide information on quality assurance methods to ensure accuracy and quality of information provided [1], if applicable.

	1	2	3	4	5	
subitem not at all important	◉	○	○	○	○	essential

Auswahl löschen

Does your paper address subitem 5-iv?

Copy and paste relevant sections from the manuscript (include quotes in quotation marks "like this" to indicate direct quotes from your manuscript), or elaborate on this item by providing additional information not in the ms, or briefly explain why the item is not applicable/relevant for your study

Meine Antwort

5-v) Ensure replicability by publishing the source code, and/or providing screenshots/screen-capture video, and/or providing flowcharts of the algorithms used

Ensure replicability by publishing the source code, and/or providing screenshots/screen-capture video, and/or providing flowcharts of the algorithms used. Replicability (i.e., other researchers should in principle be able to replicate the study) is a hallmark of scientific reporting.

	1	2	3	4	5	
subitem not at all important	◉	○	○	○	○	essential

Auswahl löschen

Does your paper address subitem 5-v?

Copy and paste relevant sections from the manuscript (include quotes in quotation marks "like this" to indicate direct quotes from your manuscript), or elaborate on this item by providing additional information not in the ms, or briefly explain why the item is not applicable/relevant for your study

Meine Antwort

5-vi) Digital preservation

Digital preservation: Provide the URL of the application, but as the intervention is likely to change or disappear over the course of the years; also make sure the intervention is archived (Internet Archive, webcitation.org, and/or publishing the source code or screenshots/videos alongside the article). As pages behind login screens cannot be archived, consider creating demo pages which are accessible without login.

	1	2	3	4	5	
subitem not at all important	○	○	◉	○	○	essential

Auswahl löschen

Does your paper address subitem 5-vi?

Copy and paste relevant sections from the manuscript (include quotes in quotation marks "like this" to indicate direct quotes from your manuscript), or elaborate on this item by providing additional information not in the ms, or briefly explain why the item is not applicable/relevant for your study

Screenshots of the intervention are provided as part of the paper and are published in the study protocol.

5-vii) Access

Access: Describe how participants accessed the application, in what setting/context, if they had to pay (or were paid) or not, whether they had to be a member of specific group. If known, describe how participants obtained "access to the platform and Internet" [1]. To ensure access for editors/reviewers/readers, consider to provide a "backdoor" login account or demo mode for reviewers/readers to explore the application (also important for archiving purposes, see vi).

	1	2	3	4	5	
subitem not at all important	○	○	◉	○	○	essential

Auswahl löschen

Does your paper address subitem 5-vii? *

Copy and paste relevant sections from the manuscript (include quotes in quotation marks "like this" to indicate direct quotes from your manuscript), or elaborate on this item by providing additional information not in the ms, or briefly explain why the item is not applicable/relevant for your study

Participants received an account and a password to access the intervention.

5-viii) Mode of delivery, features/functionalities/components of the intervention and comparator, and the theoretical framework

Describe mode of delivery, features/functionalities/components of the intervention and comparator, and the theoretical framework [6] used to design them (instructional strategy [1], behaviour change techniques, persuasive features, etc., see e.g., [7, 8] for terminology). This includes an in-depth description of the content (including where it is coming from and who developed it) [1]," whether [and how] it is tailored to individual circumstances and allows users to track their progress and receive feedback" [6]. This also includes a description of communication delivery channels and – if computer-mediated communication is a component – whether communication was synchronous or asynchronous [6]. It also includes information on presentation strategies [1], including page design principles, average amount of text on pages, presence of hyperlinks to other resources, etc. [1].

	1	2	3	4	5	
subitem not at all important	○	○	○	○	○	essential

Does your paper address subitem 5-viii? *

Copy and paste relevant sections from the manuscript (include quotes in quotation marks "like this" to indicate direct quotes from your manuscript), or elaborate on this item by providing additional information not in the ms, or briefly explain why the item is not applicable/relevant for your study

These aspects are described in the study protocol: Pischke CR, Voelcker-Rehage C, Peters M, Ratz T, Pohlabeln H, Meyer J, von Holdt K, Lippke S. Implementation and effects of information technology-based and print-based interventions to promote physical activity among community-dwelling older adults: Protocol for a randomized crossover trial. JMIR Res Protoc. 2020 Apr 27;9(4):e15168. doi: 10.2196/15168.

5-ix) Describe use parameters

Describe use parameters (e.g., intended "doses" and optimal timing for use). Clarify what instructions or recommendations were given to the user, e.g., regarding timing, frequency, heaviness of use, if any, or was the intervention used ad libitum.

	1	2	3	4	5	
subitem not at all important	○	○	○	○	◉	essential

Auswahl löschen

Does your paper address subitem 5-ix?

Copy and paste relevant sections from the manuscript (include quotes in quotation marks "like this" to indicate direct quotes from your manuscript), or elaborate on this item by providing additional information not in the ms, or briefly explain why the item is not applicable/relevant for your study

"Participants of both groups received PA recommendations according to the WHO and brochures (online and offline) were provided outlining exercises for different difficulty levels showing pictures of male vs. female older adults modelling these exercises [31] They additionally received a diary to track their PA. Depending on group assignment, all intervention material was either provided as printed material or made available on the website. The smartphone application (app) additionally provided access to the exercises and the PA diary for individuals in WEB and WEB+. On the website, in the android web-app, as well as in the printed diary, weekly feedback regarding whether PA goals were reached was provided (see figure 2 in (31)) The number of minutes or units exercised were displayed, as well as units required in order to reach the goal. The WEB+ group also used a PA tracker (Fitbit Zip, Fitbit Inc., San Francisco) in addition to the website or app, and the daily step count tracked with the device was synchronized with the website. No prompts or reminders were used on the website or in the app."

5-x) Clarify the level of human involvement

Clarify the level of human involvement (care providers or health professionals, also technical assistance) in the e-intervention or as co-intervention (detail number and expertise of professionals involved, if any, as well as "type of assistance offered, the timing and frequency of the support, how it is initiated, and the medium by which the assistance is delivered". It may be necessary to distinguish between the level of human involvement required for the trial, and the level of human involvement required for a routine application outside of a RCT setting (discuss under item 21 – generalizability).

	1	2	3	4	5	
subitem not at all important	○	○	○	○	◉	essential

Auswahl löschen

Does your paper address subitem 5-x?

Copy and paste relevant sections from the manuscript (include quotes in quotation marks "like this" to indicate direct quotes from your manuscript), or elaborate on this item by providing additional information not in the ms, or briefly explain why the item is not applicable/relevant for your study

"In tandem with the ten-week PRINT or WEB interventions, all intervention groups were offered weekly face-to-face group sessions with up to 25 participants per group which participants were encouraged to attend. The 90-min group sessions included performing the exercises in groups and going for joint walks, discussing weekly health education topics, and participants were encouraged to ask open questions regarding the exercises (also see (31)). During their first weekly group meeting, participants received the necessary equipment (printed material or access information for the website and/or a Fitbit) and a comprehensive introduction on how to use the equipment and materials. After ten weeks, group meetings were continued monthly for another six months. After the last weekly group sessions, participants chose to continue using the material of their intervention group or to start using material of one of the other groups."

5-xi) Report any prompts/reminders used

Report any prompts/reminders used: Clarify if there were prompts (letters, emails, phone calls, SMS) to use the application, what triggered them, frequency etc. It may be necessary to distinguish between the level of prompts/reminders required for the trial, and the level of prompts/reminders for a routine application outside of a RCT setting (discuss under item 21 – generalizability).

	1	2	3	4	5	
subitem not at all important	(●)	○	○	○	○	essential

Auswahl löschen

Does your paper address subitem 5-xi? *

Copy and paste relevant sections from the manuscript (include quotes in quotation marks "like this" to indicate direct quotes from your manuscript), or elaborate on this item by providing additional information not in the ms, or briefly explain why the item is not applicable/relevant for your study

Meine Antwort

5-xii) Describe any co-interventions (incl. training/support)

Describe any co-interventions (incl. training/support): Clearly state any interventions that are provided in addition to the targeted eHealth intervention, as ehealth intervention may not be designed as stand-alone intervention. This includes training sessions and support [1]. It may be necessary to distinguish between the level of training required for the trial, and the level of training for a routine application outside of a RCT setting (discuss under item 21 – generalizability.

	1	2	3	4	5	
subitem not at all important	○	○	○	○	◉	essential

Auswahl löschen

Does your paper address subitem 5-xii? *

Copy and paste relevant sections from the manuscript (include quotes in quotation marks "like this" to indicate direct quotes from your manuscript), or elaborate on this item by providing additional information not in the ms, or briefly explain why the item is not applicable/relevant for your study

"In tandem with the ten-week PRINT or WEB interventions, all intervention groups were offered weekly face-to-face group sessions with up to 25 participants per group which participants were encouraged to attend. The 90-min group sessions included performing the exercises in groups and going for joint walks, discussing weekly health education topics, and participants were encouraged to ask open questions regarding the exercises (also see (31)). During their first weekly group meeting, participants received the necessary equipment (printed material or access information for the website and/or a Fitbit) and a comprehensive introduction on how to use the equipment and materials. After ten weeks, group meetings were continued monthly for another six months. After the last weekly group sessions, participants chose to continue using the material of their intervention group or to start using material of one of the other groups."

6a) Completely defined pre-specified primary and secondary outcome measures, including how and when they were assessed

Does your paper address CONSORT subitem 6a? *

Copy and paste relevant sections from the manuscript (include quotes in quotation marks "like this" to indicate direct quotes from your manuscript), or elaborate on this item by providing additional information not in the ms, or briefly explain why the item is not applicable/relevant for your study

"The main outcomes were MVPA and SB in min per day assessed at T0, T1, and T2 via tri-axial accelerometers (GT3x+, ActiGraph, Pensacola, USA). Participants were instructed to wear the accelerometer at the right hip over the course of seven days for 24h.
Accelerometer data were processed using the Actilife 6.8.0 software and R 3.6.1 [37] was used to identify non-wear times and classify PA levels into the categories described below. Valid wear-time was derived, using the wear- and non-wear time classification algorithm by Choi et al. [38], using a 90-min window of consecutive zeros allowing a 2-min interval of nonzero-counts, and valid days were defined as having at least eight hours (480 min) of valid wear-time. There had to be at least three valid days available for each participant, including one weekend day, to be analyzed. Using one-second epochs, counts were categorized into SB (0-99 counts per min (CPM)), as well as light (0-2,690 CPM), moderate (2,691-6,166 CPM), vigorous (6167-9642 CPM), moderate-to-vigorous (2,691-9,642 CPM), and highly vigorous (>9,642 CPM) PA, according to Sasaki and colleagues [39], considering the vector magnitude.
Daily min for MVPA and SB were determined by dividing the total min by the days the accelerometer was worn. SB was additionally calculated in bouts of at least 30 min and time spent with MVPA was calculated in bouts of at least ten min. Min per week for MVPA and SB in the mentioned bouts were derived by multiplying the daily average min in 10-min/30-min bouts, respectively, by 7. Further, min of MVPA per week in bouts of ten min were dichotomized as meeting the WHO recommendation (≥ 150 minutes/week of MVPA in bouts of at least ten minutes) or not. The season during accelerometer measurement was derived from the date of examination and categorized into autumn/winter for the months of October to February and spring/summer for the months of March to September."

6a-i) Online questionnaires: describe if they were validated for online use and apply CHERRIES items to describe how the questionnaires were designed/deployed

If outcomes were obtained through online questionnaires, describe if they were validated for online use and apply CHERRIES items to describe how the questionnaires were designed/deployed [9].

	1	2	3	4	5	
subitem not at all important	◉	○	○	○	○	essential

Auswahl löschen

Does your paper address subitem 6a-i?

Copy and paste relevant sections from manuscript text

Not applicable, because outcomes were not assessed through online questionnaires.

6a-ii) Describe whether and how "use" (including intensity of use/dosage) was defined/measured/monitored

Describe whether and how "use" (including intensity of use/dosage) was defined/measured/monitored (logins, logfile analysis, etc.). Use/adoption metrics are important process outcomes that should be reported in any ehealth trial.

	1	2	3	4	5	
subitem not at all important	○	○	○	○	○	essential

Does your paper address subitem 6a-ii?

Copy and paste relevant sections from manuscript text

"Information on acceptance of the group sessions and intervention material was assessed with self-generated items (e.g., frequency of general use, use of different components (on a five-point Likert scale ranging from "never" to "daily"), and perceived helpfulness of intervention components (on five-point Likert scales ranging from "not helpful at all" to "very helpful")). Reasons for dropping out of the study, as well as for crossing or not crossing over to the respective other intervention group, and preferences for intervention material, were also assessed."

6a-iii) Describe whether, how, and when qualitative feedback from participants was obtained

Describe whether, how, and when qualitative feedback from participants was obtained (e.g., through emails, feedback forms, interviews, focus groups).

	1	2	3	4	5	
subitem not at all important	○	○	○	○	◉	essential

Auswahl löschen

Does your paper address subitem 6a-iii?

Copy and paste relevant sections from manuscript text

Qualitative feedback was obtained at the second follow-up assessment via printed questionnaires.

6b) Any changes to trial outcomes after the trial commenced, with reasons

Does your paper address CONSORT subitem 6b? *

Copy and paste relevant sections from the manuscript (include quotes in quotation marks "like this" to indicate direct quotes from your manuscript), or elaborate on this item by providing additional information not in the ms, or briefly explain why the item is not applicable/relevant for your study

No changes were made to the trial after it commenced.

7a) How sample size was determined

NPT: When applicable, details of whether and how the clustering by care provides or centers was addressed

7a-i) Describe whether and how expected attrition was taken into account when calculating the sample size

Describe whether and how expected attrition was taken into account when calculating the sample size.

	1	2	3	4	5	
subitem not at all important	○	○	○	○	◉	essential

Auswahl löschen

Does your paper address subitem 7a-i?

Copy and paste relevant sections from manuscript title (include quotes in quotation marks "like this" to indicate direct quotes from your manuscript), or elaborate on this item by providing additional information not in the ms, or briefly explain why the item is not applicable/relevant for your study

Expected attrition was taken into account while calculating the sample size, please see: Pischke CR, Voelcker-Rehage C, Peters M, Ratz T, Pohlabeln H, Meyer J, von Holdt K, Lippke S. Implementation and effects of information technology-based and print-based interventions to promote physical activity among community-dwelling older adults: Protocol for a randomized crossover trial. JMIR Res Protoc. 2020 Apr 27;9(4):e15168. doi: 10.2196/15168.

7b) When applicable, explanation of any interim analyses and stopping guidelines

Does your paper address CONSORT subitem 7b? *

Copy and paste relevant sections from the manuscript (include quotes in quotation marks "like this" to indicate direct quotes from your manuscript), or elaborate on this item by providing additional information not in the ms, or briefly explain why the item is not applicable/relevant for your study

Not applicable, no interim analyses were performed.

8a) Method used to generate the random allocation sequence

NPT: When applicable, how care providers were allocated to each trial group

Does your paper address CONSORT subitem 8a? *

Copy and paste relevant sections from the manuscript (include quotes in quotation marks "like this" to indicate direct quotes from your manuscript), or elaborate on this item by providing additional information not in the ms, or briefly explain why the item is not applicable/relevant for your study

"Weekly time slots were randomly assigned to the three intervention groups. Participants were blinded to the intervention group during randomization (i.e., they were free to choose from available time slots during the telephone interview with the study nurse without knowing which intervention group they were then assigned to)."

8b) Type of randomisation; details of any restriction (such as blocking and block size)

Does your paper address CONSORT subitem 8b? *

Copy and paste relevant sections from the manuscript (include quotes in quotation marks "like this" to indicate direct quotes from your manuscript), or elaborate on this item by providing additional information not in the ms, or briefly explain why the item is not applicable/relevant for your study

"Weekly time slots were randomly assigned to the three intervention groups. Participants were blinded to the intervention group during randomization (i.e., they were free to choose from available time slots during the telephone interview with the study nurse without knowing which intervention group they were then assigned to)."

9) Mechanism used to implement the random allocation sequence (such as sequentially numbered containers), describing any steps taken to conceal the sequence until interventions were assigned

Does your paper address CONSORT subitem 9? *

Copy and paste relevant sections from the manuscript (include quotes in quotation marks "like this" to indicate direct quotes from your manuscript), or elaborate on this item by providing additional information not in the ms, or briefly explain why the item is not applicable/relevant for your study

"Weekly time slots were randomly assigned to the three intervention groups. Participants were blinded to the intervention group during randomization (i.e., they were free to choose from available time slots during the telephone interview with the study nurse without knowing which intervention group they were then assigned to)."

10) Who generated the random allocation sequence, who enrolled participants, and who assigned participants to interventions

Does your paper address CONSORT subitem 10? *

Copy and paste relevant sections from the manuscript (include quotes in quotation marks "like this" to indicate direct quotes from your manuscript), or elaborate on this item by providing additional information not in the ms, or briefly explain why the item is not applicable/relevant for your study

Study nurses generated the random allocation sequence and assigned participants to intervention (for further information see above).

11a) If done, who was blinded after assignment to interventions (for example, participants, care providers, those assessing outcomes) and how

NPT: Whether or not administering co-interventions were blinded to group assignment

11a-i) Specify who was blinded, and who wasn't

Specify who was blinded, and who wasn't. Usually, in web-based trials it is not possible to blind the participants [1, 3] (this should be clearly acknowledged), but it may be possible to blind outcome assessors, those doing data analysis or those administering co-interventions (if any).

	1	2	3	4	5	
subitem not at all important	○	○	○	○	○	essential

Does your paper address subitem 11a-i? *

Copy and paste relevant sections from the manuscript (include quotes in quotation marks "like this" to indicate direct quotes from your manuscript), or elaborate on this item by providing additional information not in the ms, or briefly explain why the item is not applicable/relevant for your study

"Participants were not blinded to their intervention group neither were any research staff conducting the study or the statistician analyzing the data."

11a-ii) Discuss e.g., whether participants knew which intervention was the "intervention of interest" and which one was the "comparator"

Informed consent procedures (4a-ii) can create biases and certain expectations - discuss e.g., whether participants knew which intervention was the "intervention of interest" and which one was the "comparator".

	1	2	3	4	5	
subitem not at all important	○	○	◉	○	○	essential

Auswahl löschen

Does your paper address subitem 11a-ii?

Copy and paste relevant sections from the manuscript (include quotes in quotation marks "like this" to indicate direct quotes from your manuscript), or elaborate on this item by providing additional information not in the ms, or briefly explain why the item is not applicable/relevant for your study

"Participants were not blinded to their intervention group neither were any research staff conducting the study or the statistician analyzing the data."

11b) If relevant, description of the similarity of interventions

(this item is usually not relevant for ehealth trials as it refers to similarity of a placebo or sham intervention to a active medication/intervention)

Does your paper address CONSORT subitem 11b? *

Copy and paste relevant sections from the manuscript (include quotes in quotation marks "like this" to indicate direct quotes from your manuscript), or elaborate on this item by providing additional information not in the ms, or briefly explain why the item is not applicable/relevant for your study

Information regarding the similiarities of the interventions can be found here: Pischke CR, Voelcker-Rehage C, Peters M, Ratz T, Pohlabeln H, Meyer J, von Holdt K, Lippke S. Implementation and effects of information technology-based and print-based interventions to promote physical activity among community-dwelling older adults: Protocol for a randomized crossover trial. JMIR Res Protoc. 2020 Apr 27;9(4):e15168. doi: 10.2196/15168

12a) Statistical methods used to compare groups for primary and secondary outcomes

NPT: When applicable, details of whether and how the clustering by care providers or centers was addressed

Does your paper address CONSORT subitem 12a? *

Copy and paste relevant sections from the manuscript (include quotes in quotation marks "like this" to indicate direct quotes from your manuscript), or elaborate on this item by providing additional information not in the ms, or briefly explain why the item is not applicable/relevant for your study

"Observations from participants (T0, T1, T2) were excluded from the analysis sample, if they had missing information on BMI (n=14), subjective health, family status, or education (n=34) and if the MMSE score was < 13 (n=51). In total, n=501 observations from N=204 participants were included in the analysis sample.
Descriptive statistics, i.e. mean, standard deviation, range, or proportion were calculated to describe study characteristics across intervention groups and surveys. Effects of time, group, and time by group on MVPA and SB, either in average minutes per day or minutes in bouts per week were examined in multivariate linear mixed models using Linear mixed models that are capable of handling unbalanced longitudinal data with varying numbers of repeated measurements per subject [40]. Analyses were adjusted for gender, age, BMI classification, level of education, family status, employment status, household income, subjective health status, built environment, activity-related support, preference, season, and valid wear-time.
As cell-counts for cross-over groups were very small, linear mixed models were not run for the potential cross-over combinations at the 3-month follow-up. The analyses regarding intervention groups in this study were calculated using the group allocation at baseline as the indicator of intervention group (i.e., all analyses were conducted using the originally assigned groups). Numbers and proportions of individuals in the cross-over combinations are only reported descriptively. Additionally, information assessed at follow-up (e.g., on preferences, reasons for (not) crossing over to the other mode of delivery), as well as indicators of intervention adherence and acceptance, were calculated. All statistical analyses were performed using SPSS 26 [41] and SAS 9.4 [42], where the GLIMMIX procedure was used particularly for linear mixed modelling."

12a-i) Imputation techniques to deal with attrition / missing values

Imputation techniques to deal with attrition / missing values: Not all participants will use the intervention/comparator as intended and attrition is typically high in ehealth trials. Specify how participants who did not use the application or dropped out from the trial were treated in the statistical analysis (a complete case analysis is strongly discouraged, and simple imputation techniques such as LOCF may also be problematic [4]).

	1	2	3	4	5	
subitem not at all important	○	○	◉	○	○	essential

Auswahl löschen

Does your paper address subitem 12a-i? *

Copy and paste relevant sections from the manuscript (include quotes in quotation marks "like this" to indicate direct quotes from your manuscript), or elaborate on this item by providing additional information not in the ms, or briefly explain why the item is not applicable/relevant for your study

"Observations from participants (T0, T1, T2) were excluded from the analysis sample, if they had missing information on BMI (n=14), subjective health, family status, or education (n=34) and if the MMSE score was < 13 (n=51). In total, n=501 observations from N=204 participants were included in the analysis sample."

12b) Methods for additional analyses, such as subgroup analyses and adjusted analyses

Does your paper address CONSORT subitem 12b? *

Copy and paste relevant sections from the manuscript (include quotes in quotation marks "like this" to indicate direct quotes from your manuscript), or elaborate on this item by providing additional information not in the ms, or briefly explain why the item is not applicable/relevant for your study

Subgroup analyses were not performed. "Analyses were adjusted for gender, age, BMI classification, level of education, family status, employment status, household income, subjective health status, built environment, activity-related support, preference, season, and valid wear-time."

X26) REB/IRB Approval and Ethical Considerations [recommended as subheading under "Methods"] (not a CONSORT item)

X26-i) Comment on ethics committee approval

	1	2	3	4	5	
subitem not at all important	○	○	○	○	○	essential

Does your paper address subitem X26-i?

Copy and paste relevant sections from the manuscript (include quotes in quotation marks "like this" to indicate direct quotes from your manuscript), or elaborate on this item by providing additional information not in the ms, or briefly explain why the item is not applicable/relevant for your study

"Ethical approval was obtained from the Medical Association in Bremen (RA/RE-635, on July 3rd, 2018). The study was registered at the German Clinical Trials Register on January 10th, 2019 – number DRKS00016073. All study participants were fully informed about the study and were requested to give informed consent."

x26-ii) Outline informed consent procedures

Outline informed consent procedures e.g., if consent was obtained offline or online (how? Checkbox, etc.?), and what information was provided (see 4a-ii). See [6] for some items to be included in informed consent documents.

	1	2	3	4	5	
subitem not at all important	○	○	○	○	◉	essential

Auswahl löschen

Does your paper address subitem X26-ii?

Copy and paste relevant sections from the manuscript (include quotes in quotation marks "like this" to indicate direct quotes from your manuscript), or elaborate on this item by providing additional information not in the ms, or briefly explain why the item is not applicable/relevant for your study

Consent was obtained offline. "Ethical approval was obtained from the Medical Association in Bremen (RA/RE-635, on July 3rd, 2018). The study was registered at the German Clinical Trials Register on January 10th, 2019 – number DRKS00016073. All study participants were fully informed about the study and were requested to give informed consent."

X26-iii) Safety and security procedures

Safety and security procedures, incl. privacy considerations, and any steps taken to reduce the likelihood or detection of harm (e.g., education and training, availability of a hotline)

	1	2	3	4	5	
subitem not at all important	(●)	( )	( )	( )	( )	essential

Auswahl löschen

Does your paper address subitem X26-iii?

Copy and paste relevant sections from the manuscript (include quotes in quotation marks "like this" to indicate direct quotes from your manuscript), or elaborate on this item by providing additional information not in the ms, or briefly explain why the item is not applicable/relevant for your study

Meine Antwort

RESULTS

13a) For each group, the numbers of participants who were randomly assigned, received intended treatment, and were analysed for the primary outcome

NPT: The number of care providers or centers performing the intervention in each group and the number of patients treated by each care provider in each center

Does your paper address CONSORT subitem 13a? *

Copy and paste relevant sections from the manuscript (include quotes in quotation marks "like this" to indicate direct quotes from your manuscript), or elaborate on this item by providing additional information not in the ms, or briefly explain why the item is not applicable/relevant for your study

"Of the initially recruited n=242 participants, n=195 participants completed T1 (original group allocation; WEB: n=74, WEB+: n=30, PRINT: n=91). After T1, n=179 chose to remain in their previous intervention group and n=16 decided to cross over to the other group. Finally, n=160 participants completed T2 (original group allocation; WEB: n=59, IT+: n=22, PRINT: n=79). Attrition rates from baseline to T2 were 33.9% across groups (35.2% in WEB, 42.1% in WEB+, and 30.1% in PRINT)."

13b) For each group, losses and exclusions after randomisation, together with reasons

Does your paper address CONSORT subitem 13b? (NOTE: Preferably, this is shown in a CONSORT flow diagram) *

Copy and paste relevant sections from the manuscript (include quotes in quotation marks "like this" to indicate direct quotes from your manuscript), or elaborate on this item by providing additional information not in the ms, or briefly explain why the item is not applicable/relevant for your study

"The analysis sample (n=204) did not differ from the recruited sample (n=242) in terms of baseline characteristics, as the effect sizes (Cohen's d and Cohen's h, respectively) were all <.20. The only exception was cognitive state: The analysis sample had a slightly higher MMSE-2-BV mean score compared to the recruited sample (Cohen's d = .22). This was expected due to the exclusion criteria (see Figure 1)."

13b-i) Attrition diagram

Strongly recommended: An attrition diagram (e.g., proportion of participants still logging in or using the intervention/comparator in each group plotted over time, similar to a survival curve) or other figures or tables demonstrating usage/dose/engagement.

	1	2	3	4	5	
subitem not at all important	O	O	O	O	O	essential

Does your paper address subitem 13b-i?

Copy and paste relevant sections from the manuscript or cite the figure number if applicable (include quotes in quotation marks "like this" to indicate direct quotes from your manuscript), or elaborate on this item by providing additional information not in the ms, or briefly explain why the item is not applicable/relevant for your study

An attrition diagram is included as figure 1.

14a) Dates defining the periods of recruitment and follow-up

Does your paper address CONSORT subitem 14a? *

Copy and paste relevant sections from the manuscript (include quotes in quotation marks "like this" to indicate direct quotes from your manuscript), or elaborate on this item by providing additional information not in the ms, or briefly explain why the item is not applicable/relevant for your study

Dates defining the start and end dates of recruitment and follow-up can be looked up at the German Clinical Trials Register on January 10th, 2019 – number DRKS00016073.

14a-i) Indicate if critical "secular events" fell into the study period

Indicate if critical "secular events" fell into the study period, e.g., significant changes in Internet resources available or "changes in computer hardware or Internet delivery resources"

	1	2	3	4	5	
subitem not at all important	(●)	○	○	○	○	essential

Auswahl löschen

Does your paper address subitem 14a-i?

Copy and paste relevant sections from the manuscript (include quotes in quotation marks "like this" to indicate direct quotes from your manuscript), or elaborate on this item by providing additional information not in the ms, or briefly explain why the item is not applicable/relevant for your study

Meine Antwort

14b) Why the trial ended or was stopped (early)

Does your paper address CONSORT subitem 14b? *

Copy and paste relevant sections from the manuscript (include quotes in quotation marks "like this" to indicate direct quotes from your manuscript), or elaborate on this item by providing additional information not in the ms, or briefly explain why the item is not applicable/relevant for your study

The trial did not end or stop early.

15) A table showing baseline demographic and clinical characteristics for each group

NPT: When applicable, a description of care providers (case volume, qualification, expertise, etc.) and centers (volume) in each group

Does your paper address CONSORT subitem 15? *

Copy and paste relevant sections from the manuscript (include quotes in quotation marks "like this" to indicate direct quotes from your manuscript), or elaborate on this item by providing additional information not in the ms, or briefly explain why the item is not applicable/relevant for your study

"Baseline demographic characteristics of participants included in the analysis sample are displayed in Table 2."

15-i) Report demographics associated with digital divide issues

In ehealth trials it is particularly important to report demographics associated with digital divide issues, such as age, education, gender, social-economic status, computer/Internet/ehealth literacy of the participants, if known.

	1	2	3	4	5	
subitem not at all important	○	○	◉	○	○	essential

Auswahl löschen

Does your paper address subitem 15-i? *

Copy and paste relevant sections from the manuscript (include quotes in quotation marks "like this" to indicate direct quotes from your manuscript), or elaborate on this item by providing additional information not in the ms, or briefly explain why the item is not applicable/relevant for your study

"Baseline demographic characteristics of participants included in the analysis sample are displayed in Table 2."

16) For each group, number of participants (denominator) included in each analysis and whether the analysis was by original assigned groups

16-i) Report multiple "denominators" and provide definitions

Report multiple "denominators" and provide definitions: Report N's (and effect sizes) "across a range of study participation [and use] thresholds" [1], e.g., N exposed, N consented, N used more than x times, N used more than y weeks, N participants "used" the intervention/comparator at specific pre-defined time points of interest (in absolute and relative numbers per group). Always clearly define "use" of the intervention.

	1	2	3	4	5	
subitem not at all important	◉	○	○	○	○	essential

Auswahl löschen

Does your paper address subitem 16-i? *

Copy and paste relevant sections from the manuscript (include quotes in quotation marks "like this" to indicate direct quotes from your manuscript), or elaborate on this item by providing additional information not in the ms, or briefly explain why the item is not applicable/relevant for your study

Meine Antwort

16-ii) Primary analysis should be intent-to-treat

Primary analysis should be intent-to-treat, secondary analyses could include comparing only "users", with the appropriate caveats that this is no longer a randomized sample (see 18-i).

	1	2	3	4	5	
subitem not at all important	◉	○	○	○	○	essential

Auswahl löschen

Does your paper address subitem 16-ii?

Copy and paste relevant sections from the manuscript (include quotes in quotation marks "like this" to indicate direct quotes from your manuscript), or elaborate on this item by providing additional information not in the ms, or briefly explain why the item is not applicable/relevant for your study

Meine Antwort

17a) For each primary and secondary outcome, results for each group, and the estimated effect size and its precision (such as 95% confidence interval)

Does your paper address CONSORT subitem 17a? *

Copy and paste relevant sections from the manuscript (include quotes in quotation marks "like this" to indicate direct quotes from your manuscript), or elaborate on this item by providing additional information not in the ms, or briefly explain why the item is not applicable/relevant for your study

Please see table 3 for results of each group, estimated effect size and its precision.

17a-i) Presentation of process outcomes such as metrics of use and intensity of use

In addition to primary/secondary (clinical) outcomes, the presentation of process outcomes such as metrics of use and intensity of use (dose, exposure) and their operational definitions is critical. This does not only refer to metrics of attrition (13-b) (often a binary variable), but also to more continuous exposure metrics such as "average session length". These must be accompanied by a technical description how a metric like a "session" is defined (e.g., timeout after idle time) [1] (report under item 6a).

	1	2	3	4	5	
subitem not at all important	○	○	◉	○	○	essential

Auswahl löschen

Does your paper address subitem 17a-i?

Copy and paste relevant sections from the manuscript (include quotes in quotation marks "like this" to indicate direct quotes from your manuscript), or elaborate on this item by providing additional information not in the ms, or briefly explain why the item is not applicable/relevant for your study

"Overall, attendance of the face-to-face components of the intervention was high with an average of 8/10 attending the weekly group sessions and 2/3 attending the monthly group sessions (see Table 5/supplement). With regard to use of the PA diary, there were no marked differences between the intervention groups at T1. The exercise brochure was used at least once per week by 68.5% in PRINT, 45.3% in WEB and 38.4% in WEB+. Overall use of intervention material was high to moderate at T1 (PA diary was used by between 65% and 80% at least once per week) and declined by T2. At T2, approximately 44% in PRINT, 49% in WEB, and 58% in WEB+ still used the PA diary at least once per week. Use of the smartphone app was very low in WEB but higher in WEB+."

17b) For binary outcomes, presentation of both absolute and relative effect sizes is recommended

Does your paper address CONSORT subitem 17b? *

Copy and paste relevant sections from the manuscript (include quotes in quotation marks "like this" to indicate direct quotes from your manuscript), or elaborate on this item by providing additional information not in the ms, or briefly explain why the item is not applicable/relevant for your study

Not applicable, binary outcomes were not included.

18) Results of any other analyses performed, including subgroup analyses and adjusted analyses, distinguishing pre-specified from exploratory

Does your paper address CONSORT subitem 18? *

Copy and paste relevant sections from the manuscript (include quotes in quotation marks "like this" to indicate direct quotes from your manuscript), or elaborate on this item by providing additional information not in the ms, or briefly explain why the item is not applicable/relevant for your study

No subgroup analyses were performed but "analyses were adjusted for gender, age, BMI classification, level of education, family status, employment status, household income, subjective health status, built environment, activity-related support, preference, season, and valid wear-time."

18-i) Subgroup analysis of comparing only users

A subgroup analysis of comparing only users is not uncommon in ehealth trials, but if done, it must be stressed that this is a self-selected sample and no longer an unbiased sample from a randomized trial (see 16-iii).

	1	2	3	4	5	
subitem not at all important	◉	○	○	○	○	essential

Auswahl löschen

Does your paper address subitem 18-i?

Copy and paste relevant sections from the manuscript (include quotes in quotation marks "like this" to indicate direct quotes from your manuscript), or elaborate on this item by providing additional information not in the ms, or briefly explain why the item is not applicable/relevant for your study

Meine Antwort

19) All important harms or unintended effects in each group

(for specific guidance see CONSORT for harms)

Does your paper address CONSORT subitem 19? *

Copy and paste relevant sections from the manuscript (include quotes in quotation marks "like this" to indicate direct quotes from your manuscript), or elaborate on this item by providing additional information not in the ms, or briefly explain why the item is not applicable/relevant for your study

There were no harms or unintended effects.

19-i) Include privacy breaches, technical problems

Include privacy breaches, technical problems. This does not only include physical "harm" to participants, but also incidents such as perceived or real privacy breaches [1], technical problems, and other unexpected/unintended incidents. "Unintended effects" also includes unintended positive effects [2].

	1	2	3	4	5	
subitem not at all important	◉	○	○	○	○	essential

Auswahl löschen

Does your paper address subitem 19-i?

Copy and paste relevant sections from the manuscript (include quotes in quotation marks "like this" to indicate direct quotes from your manuscript), or elaborate on this item by providing additional information not in the ms, or briefly explain why the item is not applicable/relevant for your study

There were no privacy breaches or technical problems.

19-ii) Include qualitative feedback from participants or observations from staff/researchers

Include qualitative feedback from participants or observations from staff/researchers, if available, on strengths and shortcomings of the application, especially if they point to unintended/unexpected effects or uses. This includes (if available) reasons for why people did or did not use the application as intended by the developers.

	1	2	3	4	5	
subitem not at all important	O	O	O	O	O	essential

Does your paper address subitem 19-ii?

Copy and paste relevant sections from the manuscript (include quotes in quotation marks "like this" to indicate direct quotes from your manuscript), or elaborate on this item by providing additional information not in the ms, or briefly explain why the item is not applicable/relevant for your study

"Acceptance of the interventions was generally high; approximately 90% agreed that the program was at least somewhat helpful for being physically active. Also, approximately 90% stated that they would recommend it to others. High satisfaction and acceptance seemed to be irrespective of the delivery mode. Retrospectively, between 70% and 80% in each group stated that their random allocation matched their initial preference (see Table 5/supplement). The reasons for crossing over most commonly reported were "wanted to try something new" (n=9), "liked the website and wanted to use the fitness tracker in addition" (n=7), and "high affinity to technology" (n=5). The reason for not crossing over reported most often was "completely satisfied with the current material" (n=84). Further reasons listed included "did not want to lose contact to the previous group members" (n=21), "printed version seemed impractical" (n=14), or "not technology-affine and wanted to keep the printed version" (n=9, data not shown). No unintended effects were reported by participants."

DISCUSSION

22) Interpretation consistent with results, balancing benefits and harms, and considering other relevant evidence

NPT: In addition, take into account the choice of the comparator, lack of or partial blinding, and unequal expertise of care providers or centers in each group

22-i) Restate study questions and summarize the answers suggested by the data, starting with primary outcomes and process outcomes (use)

Restate study questions and summarize the answers suggested by the data, starting with primary outcomes and process outcomes (use).

	1	2	3	4	5	
subitem not at all important	O	O	O	O	O	essential

Does your paper address subitem 22-i? *

Copy and paste relevant sections from the manuscript (include quotes in quotation marks "like this" to indicate direct quotes from your manuscript), or elaborate on this item by providing additional information not in the ms, or briefly explain why the item is not applicable/relevant for your study

"In sum, no intervention effects on MVPA could be detected in this study including 242 community-dwelling older adults aged 60 years and above participated in a nine-month randomized trial with a cross-over design. MVPA did not increase, but decreased over time, regardless of the group participants were randomized to. The proportion of participants reaching the WHO recommendations for MVPA remained relatively stable with approximately 20% meeting the recommendations at all three assessment points. The use of intervention materials decreased slightly over time. Regarding SB, all three intervention groups displayed decreases in this risk behavior over time. However, no significant group differences were noted. Interestingly, however, there was an indication that the reduction was most pronounced in the WEB-group which decreased sedentary time in 30-min bouts in min per week from baseline to T1. However, this effect was not maintained at follow-up and no significant time by group interactions could be observed.
It is conceivable that intervention effects on MVPA are masked due to latent (unobserved) heterogeneous subgroups. When analyzing trajectories of MVPA and SB [44], several subgroups of participants could be identified in our sample with differing trajectories. There were two groups remaining constant in their activity and inactivity levels (active/ inactive at all three time points). In addition, there was a third group of individuals that started out with high levels of SB at baseline and decreased SB and increased MVPA significantly over time. This group also displayed significantly higher levels of baseline action self-efficacy (Ratz et al., under review, [44]). This may suggest that our interventions were particularly useful for initially inactive adults with high levels of SB.
Secondly, we found that, despite having had the opportunity to try out another condition at six-month follow-up, only very few participants took advantage of it. In total, only approximately seven percent (n=16) switched condition at follow-up (n=1 from WEB to PRINT, n=15 from PRINT to WEB). Three thirds of participants in each condition stated at follow-up that their random allocation had matched their initial preference suggesting that intervention participants felt content with the condition (and use of materials) that they were assigned to. In addition, feeling part of a group during sessions and not wanting to leave the group may have played a role. Twenty-one participants stated that they did not want to lose contact with their group. In fact, overall satisfaction with the group sessions and rates of attendance were very high and did not differ between mode of delivery. To conclude, these results suggest that participants did not see any reason for switching to another mode of delivery. However, another way of explaining the lack of movement between conditions at T1 could be that study participants who were randomized to a specific condition either refused to participate in the study or possibly dropped out of the study early, because they felt dissatisfied with using the intervention materials assigned to them in the condition that they were randomized to. Future pragmatic trials combining a randomized controlled trial with two different intervention arms that participants can self-select to will be necessary to further investigate the questions regarding the role of individual preferences raised in this study. Another question that remains is whether the recruitment channel that participants were recruited via (i.e., print media vs. mailed invitations) played a role when deciding for or against an intervention condition at the six-month follow-up. This will be the topic of a subsequent article."

22-ii) Highlight unanswered new questions, suggest future research

Highlight unanswered new questions, suggest future research.

	1	2	3	4	5	
subitem not at all important	O	O	O	O	O	essential

Does your paper address subitem 22-ii?

Copy and paste relevant sections from the manuscript (include quotes in quotation marks "like this" to indicate direct quotes from your manuscript), or elaborate on this item by providing additional information not in the ms, or briefly explain why the item is not applicable/relevant for your study

"Further, it is known from previous research that preferences for intervention modality may vary by age, gender, BMI or by social or living environment [22-24]. Younger individuals seem to prefer eHealth over print-based interventions [23-24]), whereas older or female individuals or those with an adverse weight status appear to be more likely to be in favor of print-based interventions [23]. Unfortunately, in our study, we were not able to investigate variations by socio-demographic characteristics because only seven percent of the sample changed groups at follow-up and preference was not assessed at baseline, but only retrospectively."

20) Trial limitations, addressing sources of potential bias, imprecision, and, if relevant, multiplicity of analyses

20-i) Typical limitations in ehealth trials

Typical limitations in ehealth trials: Participants in ehealth trials are rarely blinded. Ehealth trials often look at a multiplicity of outcomes, increasing risk for a Type I error. Discuss biases due to non-use of the intervention/usability issues, biases through informed consent procedures, unexpected events.

	1	2	3	4	5	
subitem not at all important	O	O	O	O	O	essential

Does your paper address subitem 20-i? *

Copy and paste relevant sections from the manuscript (include quotes in quotation marks "like this" to indicate direct quotes from your manuscript), or elaborate on this item by providing additional information not in the ms, or briefly explain why the item is not applicable/relevant for your study

"Despite the advantages of the study design applied in this project and the objective measurement of MVPA using accelerometers, this study had several limitations. Firstly, there was no untreated or placebo control group. Secondly, the target population consisted of rather active older adults and individuals who had recently become regularly physically active. Thirdly, preference for a certain intervention delivery mode at baseline was only assessed retrospectively which may have led to recall bias. However, we chose to assess preferences retrospectively because we anticipated disappointment (and possibly study drop-out), if a person was not randomized to the intervention group they preferred to be in at baseline. Finally, we were not able to recruit the number of study participants to the study that we had aimed for [31] and loss-to-follow-up was relatively high. As a result of not meeting the intended goal regarding sample size, our analyses were underpowered. This problem was dealt with by using linear mixed modelling. Another limitation of our study is that the primary outcome defined in this study was not a state of the art recommendation at the time of completion of the study. Thus, participants may not have been sufficiently motivated to engage in activities amounting to fewer than ten min because, on the website or using the PRINT materials for self-monitoring PA, they could only complete the PA diary, if the activities leveled-up to 10-min bouts. Lastly, we were not able to quantify the individual intervention effect of the group sessions on PA."

21) Generalisability (external validity, applicability) of the trial findings

NPT: External validity of the trial findings according to the intervention, comparators, patients, and care providers or centers involved in the trial

21-i) Generalizability to other populations

Generalizability to other populations: In particular, discuss generalizability to a general Internet population, outside of a RCT setting, and general patient population, including applicability of the study results for other organizations

	1	2	3	4	5	
subitem not at all important	○	○	○	○	○	essential

Does your paper address subitem 21-i?

Copy and paste relevant sections from the manuscript (include quotes in quotation marks "like this" to indicate direct quotes from your manuscript), or elaborate on this item by providing additional information not in the ms, or briefly explain why the item is not applicable/relevant for your study

Generalizability is limted because "the target population consisted of rather active older adults and individuals who had recently become regularly physically active."

21-ii) Discuss if there were elements in the RCT that would be different in a routine application setting

Discuss if there were elements in the RCT that would be different in a routine application setting (e.g., prompts/reminders, more human involvement, training sessions or other co-interventions) and what impact the omission of these elements could have on use, adoption, or outcomes if the intervention is applied outside of a RCT setting.

	1	2	3	4	5	
subitem not at all important	◉	○	○	○	○	essential

Auswahl löschen

Does your paper address subitem 21-ii?

Copy and paste relevant sections from the manuscript (include quotes in quotation marks "like this" to indicate direct quotes from your manuscript), or elaborate on this item by providing additional information not in the ms, or briefly explain why the item is not applicable/relevant for your study

Meine Antwort

OTHER INFORMATION

23) Registration number and name of trial registry

Does your paper address CONSORT subitem 23? *

Copy and paste relevant sections from the manuscript (include quotes in quotation marks "like this" to indicate direct quotes from your manuscript), or elaborate on this item by providing additional information not in the ms, or briefly explain why the item is not applicable/relevant for your study

"The study was registered at the German Clinical Trials Register on January 10th, 2019 – number DRKS00016073."

24) Where the full trial protocol can be accessed, if available

Does your paper address CONSORT subitem 24? *

Cite a Multimedia Appendix, other reference, or copy and paste relevant sections from the manuscript (include quotes in quotation marks "like this" to indicate direct quotes from your manuscript), or elaborate on this item by providing additional information not in the ms, or briefly explain why the item is not applicable/relevant for your study

Pischke CR, Voelcker-Rehage C, Peters M, Ratz T, Pohlabeln H, Meyer J, von Holdt K, Lippke S. Implementation and effects of information technology-based and print-based interventions to promote physical activity among community-dwelling older adults: Protocol for a randomized crossover trial. JMIR Res Protoc. 2020 Apr 27;9(4):e15168. doi: 10.2196/15168.

25) Sources of funding and other support (such as supply of drugs), role of funders

Does your paper address CONSORT subitem 25? *

Copy and paste relevant sections from the manuscript (include quotes in quotation marks "like this" to indicate direct quotes from your manuscript), or elaborate on this item by providing additional information not in the ms, or briefly explain why the item is not applicable/relevant for your study

"This study is funded by the German Federal Ministry of Education and Research (BMBF; project numbers 01EL1822A, 01EL1822F, 01EL1822I, 01EL1822C). The content of this article only reflects the authors' views and the funder is not liable for any use that may be made of the information contained therein."

X27) Conflicts of Interest (not a CONSORT item)

X27-i) State the relation of the study team towards the system being evaluated

In addition to the usual declaration of interests (financial or otherwise), also state the relation of the study team towards the system being evaluated, i.e., state if the authors/evaluators are distinct from or identical with the developers/sponsors of the intervention.

	1	2	3	4	5	
subitem not at all important	○	○	○	○	○	essential

Does your paper address subitem X27-i?

Copy and paste relevant sections from the manuscript (include quotes in quotation marks "like this" to indicate direct quotes from your manuscript), or elaborate on this item by providing additional information not in the ms, or briefly explain why the item is not applicable/relevant for your study

The authors declare that they have no competing interests. Two of the authors created the intervention.

About the CONSORT EHEALTH checklist

As a result of using this checklist, did you make changes in your manuscript? *

- ○ yes, major changes
- ○ yes, minor changes
- ◉ no

What were the most important changes you made as a result of using this checklist?

Meine Antwort

How much time did you spend on going through the checklist INCLUDING making changes in your manuscript *

We spent 90 minutes going through the checklist.

As a result of using this checklist, do you think your manuscript has improved? *

- (x) yes
- ( ) no
- ( ) Sonstiges:

Would you like to become involved in the CONSORT EHEALTH group?

This would involve for example becoming involved in participating in a workshop and writing an "Explanation and Elaboration" document

- (x) yes
- ( ) no
- ( ) Sonstiges:

Auswahl löschen

Any other comments or questions on CONSORT EHEALTH

Meine Antwort

STOP - Save this form as PDF before you click submit

To generate a record that you filled in this form, we recommend to generate a PDF of this page (on a Mac, simply select "print" and then select "print as PDF") before you submit it.

When you submit your (revised) paper to JMIR, please upload the PDF as supplementary file.

Don't worry if some text in the textboxes is cut off, as we still have the complete information in our database. Thank you!

Final step: Click submit !

Click submit so we have your answers in our database!

Senden

Geben Sie niemals Passwörter über Google Formulare weiter.

Dieser Inhalt wurde nicht von Google erstellt und wird von Google auch nicht unterstützt. Missbrauch melden - Nutzungsbedingungen - Datenschutzerklärung

Google Formulare
